# *Cdon* Mutation and Fetal Ethanol Exposure Synergize to Produce Midline Signaling Defects and Holoprosencephaly Spectrum Disorders in Mice

**DOI:** 10.1371/journal.pgen.1002999

**Published:** 2012-10-11

**Authors:** Mingi Hong, Robert S. Krauss

**Affiliations:** Department of Developmental and Regenerative Biology, Mount Sinai School of Medicine, New York, New York, United States of America; Albert Einstein College of Medicine, United States of America

## Abstract

Holoprosencephaly (HPE) is a remarkably common congenital anomaly characterized by failure to define the midline of the forebrain and midface. HPE is associated with heterozygous mutations in Sonic hedgehog (SHH) pathway components, but clinical presentation is extremely variable and many mutation carriers are unaffected. It has been proposed that these observations are best explained by a multiple-hit model, in which the penetrance and expressivity of an HPE mutation is enhanced by a second mutation or the presence of cooperating, but otherwise silent, modifier genes. Non-genetic risk factors are also implicated in HPE, and gene–environment interactions may provide an alternative multiple-hit model to purely genetic multiple-hit models; however, there is little evidence for this contention. We report here a mouse model in which there is dramatic synergy between mutation of a bona fide HPE gene (*Cdon*, which encodes a SHH co-receptor) and a suspected HPE teratogen, ethanol. Loss of *Cdon* and in utero ethanol exposure in 129S6 mice give little or no phenotype individually, but together produce defects in early midline patterning, inhibition of SHH signaling in the developing forebrain, and a broad spectrum of HPE phenotypes. Our findings argue that ethanol is indeed a risk factor for HPE, but genetically predisposed individuals, such as those with SHH pathway mutations, may be particularly susceptible. Furthermore, gene–environment interactions are likely to be important in the multifactorial etiology of HPE.

## Author Summary

Holoprosencephaly (HPE), a congenital anomaly characterized by failure to form the midline of the forebrain and midface, occurs as frequently as 1 in 250 conceptions. Mutations in genes that direct formation of the forebrain and facial midline are associated with HPE, but the clinical outcome is extremely variable and many mutation carriers are unaffected. This has led to the hypothesis that more than one genetic insult is required to cause HPE. Although multiple mutations have been identified in some individuals with HPE, they represent a small percentage of the total. Non-genetic risk factors are also implicated in HPE, and it is possible that this defect may arise as a consequence of a mutation interacting with an environmental exposure. However, evidence for this possibility is lacking. We have developed a mouse HPE model in which there is dramatic synergy between mutation in the *Cdon* gene, which is mutated in some HPE patients, and in utero exposure to ethanol, a suspected but unproven HPE risk factor. Our findings argue that ethanol is a risk factor for HPE, but genetically predisposed individuals may be particularly susceptible. Furthermore, gene–environment interactions are likely to be important in the multifactorial etiology of HPE.

## Introduction

Holoprosencephaly (HPE) is a congenital anomaly characterized by failure to define the midline of the forebrain and midface [1]. HPE occurs with the remarkable frequency of ∼1∶250 conceptions but, due to intrauterine lethality, live-born prevalence is ∼1∶10,000 [2], [3]. A phenotypic continuum of HPE defects is broadly classified into three categories based on the degree of midline cleavage of the forebrain [4], [5]. Alobar HPE, the most severe form, is characterized by complete failure to partition the forebrain into left and right hemispheres; semilobar and lobar HPE are progressively less severe subtypes. The spectrum of craniofacial midline anomalies ranges from cyclopia in the most severe cases to single nostril, midface hypoplasia with cleft lip and/or palate, hypotelorism (abnormally close-set eyes) and solitary median maxillary central incisor in progressively less affected individuals. Mild facial midline abnormalities, called HPE microforms, can occur in the absence of brain malformations.

The etiology of HPE is heterogeneous, with genetic and environmental factors implicated [5]–[7]. Heterozygous mutations in Sonic Hedgehog (SHH) pathway components are found in both inherited and sporadic HPE, including SHH itself, the primary receptor PTCH1, the co-receptors CDON (also called CDO) and GAS1, and the transcription factor, GLI2 [6], [8]–[11]. HPE is characterized by extensive phenotypic variability; this variability is seen in both sporadic cases and within pedigrees [12]. As many as one-third of mutation carriers in pedigrees do not exhibit a clinical phenotype, and mutations found in many sporadic HPE patients are inherited from unaffected parents [12], [13]. These observations strongly suggest that heterozygous mutation of HPE genes is insufficient to produce severe anomalies and have led to the proposal that HPE is a multiple-hit disorder which arises from a complex interplay of developmental, genetic (both mutations and more common modifier alleles) and environmental factors [5]–[7], [14]. Consistent with this notion, HPE cases with double genetic variations were recently reported, including individuals in which one mutation was inherited and the other arose de novo [13].

In addition to the evidence for multiple-genetic-hit models of HPE, it has been hypothesized that gene-environment interactions may be involved, with a synergistic combination of genetic and non-genetic insults, but there is little or no direct evidence for this. Epidemiological studies suggest that preexisting maternal diabetes and fetal alcohol exposure are potential non-genetic risk factors for HPE, but such studies have been difficult because they rely on birth prevalence not overall prevalence, resulting in small sample sizes and, consequently, wide confidence intervals and interstudy variability [15], [16]. A question of public health relevance is whether genetic predisposition to HPE (e.g., heterozygous mutation of a SHH pathway component) sensitizes individuals to environmental agents. The challenges faced by HPE epidemiology suggest that the ability to assess gene-environment interactions in patient cohorts might be impracticable and that animal models are required to address this point [15], [16]. Although fetal alcohol exposure has been implicated in HPE in animal models, results with the mouse, the model organism that best mirrors human genetic susceptibility to HPE, have been inconsistent, with most strains resistant to ethanol and sensitive strains showing low penetrance of HPE phenotypes [17]–[22].

CDON and BOC are related, cell surface SHH-binding proteins that promote SHH pathway activity as co-receptors with PTCH1 [23]–[28]. We have recently identified heterozygous, loss-of-function *CDON* mutations in HPE patients, including at least one that arose de novo [9]. However, deletions of one copy of *CDON* have also been identified in individuals without overt HPE phenotypes [29]. These findings are consistent with the notion that additional events, genetic or environmental, may be required for production of HPE in *CDON* mutation carriers. Studies with mice support this concept. *Cdon^−/−^* mice display HPE with strain-dependent severity [28], [30]. *Cdon^−/−^* mice on a C57BL/6NTac background have semi-lobar HPE with a single nostril with high penetrance, whereas these mice on a 129S6/SvEvTac background (129S6.*Cdon^−/−^* mice) show only HPE microforms with low penetrance. Although *Shh^+/−^* mice and *Boc^−/−^* mice do not have HPE, removal of one copy of *Shh* or gene dosage-dependent removal of *Boc* from 129S6.*Cdon^−/−^* mice results in much more severe HPE phenotypes [25], [27]. These results suggest that 129S6.*Cdon^−/−^* mice have a largely subthreshold defect in SHH signaling that renders them sensitive to second hits, and they are useful as a model for the multifactorial nature of HPE [25], [27]. 129S6 mice are resistant to ethanol teratogenesis [18]. In this study, we therefore tested whether *Cdon^−/−^* mice of this strain are sensitized to ethanol-induced HPE. We report that loss of CDON and in utero ethanol exposure in 129S6 mice results in synergistic and specific defects in early midline patterning, inhibition of SHH signaling in the rostroventral midline, and a broad spectrum of HPE phenotypes. Therefore, loss of *Cdon* is sufficient to confer sensitivity to ethanol-induced HPE. Our findings argue that ethanol is indeed a risk factor for HPE, but genetically predisposed individuals, such as those with SHH pathway mutations, may be particularly susceptible. Furthermore, gene-environment interactions are likely to be involved in the multifactorial etiology of HPE.

## Results

### *Cdon* mutation and fetal ethanol exposure synergize to produce HPE in 129S6 mice

We adapted a protocol for *in utero* ethanol exposure used for studies on fetal alcohol spectrum disorders [31], [32] to assess whether 129S6.*Cdon^−/−^* mice were sensitized to ethanol-induced HPE (see Materials and Methods, Figure S1 and Table S1). All studies were performed with mice on the 129S6 background, and animals are referred to only by genotype unless otherwise noted. *Cdon^+/−^* mice were intercrossed and pregnant females received IP injections of ethanol or saline control at E7.0 and 4 hours later (when embryos are at the gastrulation stage). At E8.0, ethanol-treated embryos had, on average, between one and two fewer somite pairs than saline-treated controls, regardless of genotype; ethanol-treated embryos at E9.0 and E10.0 had a similar deficit in somite numbers despite having many more somites at these stages, indicating that ethanol induced an early, transient developmental delay of approximately two to four hr that was independent of *Cdon* status (Table S2). Embryos were then assessed for HPE phenotypes between E10.0 and E19.0. *Cdon^+/+^* and *Cdon^+/−^* embryos with or without ethanol did not have HPE, and saline-treated *Cdon^−/−^* embryos showed only microform HPE at low penetrance, similar to untreated *Cdon^−/−^* embryos. In contrast, ∼75% of ethanol-exposed E10.0–E19.0 *Cdon^−/−^* embryos displayed HPE-related phenotypes of varying severity. Therefore, mutation of *Cdon* and fetal ethanol exposure synergized to produce the HPE spectrum.

**Figure 1:**
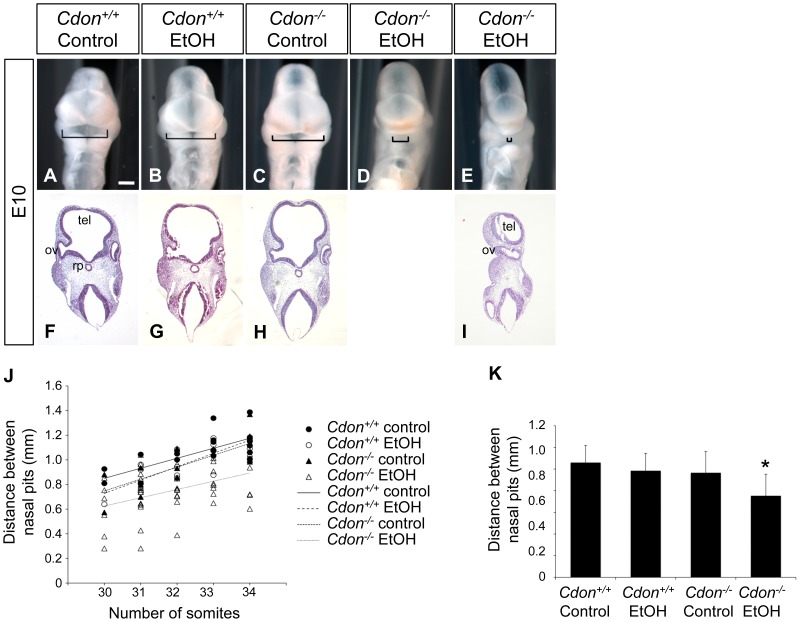
Synergy between loss of *Cdon* and fetal ethanol exposure produces HPE: E10.0. (A–E) Frontal views of E10.0 embryos of the indicated genotype treated in utero with ethanol (EtOH) or saline (control). Brackets indicate distance between the left and right nasal pits, a measure of midline formation. Scale bar, 250 µm. (F–I) Coronal sections stained with H&E. Some EtOH-treated *Cdon^−/−^* embryos (E, I) displayed severe HPE with loss of telencephalic structure (tel), failure to divide the optic vesicle (ov), and absence of Rathke's pouch (rp). (J) Quantification of the distance between the nasal pits in control and ethanol-treated *Cdon^+/+^* and *Cdon^−/−^* embryos. Embryos were harvested at E10.0 and those between 30–34 somites were scored. Distance between the nasal pits was measured for each embryo and plotted against somite number. (K) The data shown in (J) displayed as means ± SD. *Cdon^+/+^* (Control), n = 19; *Cdon^+/+^*(EtOH), n = 17; *Cdon^−/−^*(Control), n = 18; *Cdon^−/−^*(EtOH), n = 51. *, p<0.001 compared to the three other conditions.

**Figure 2:**
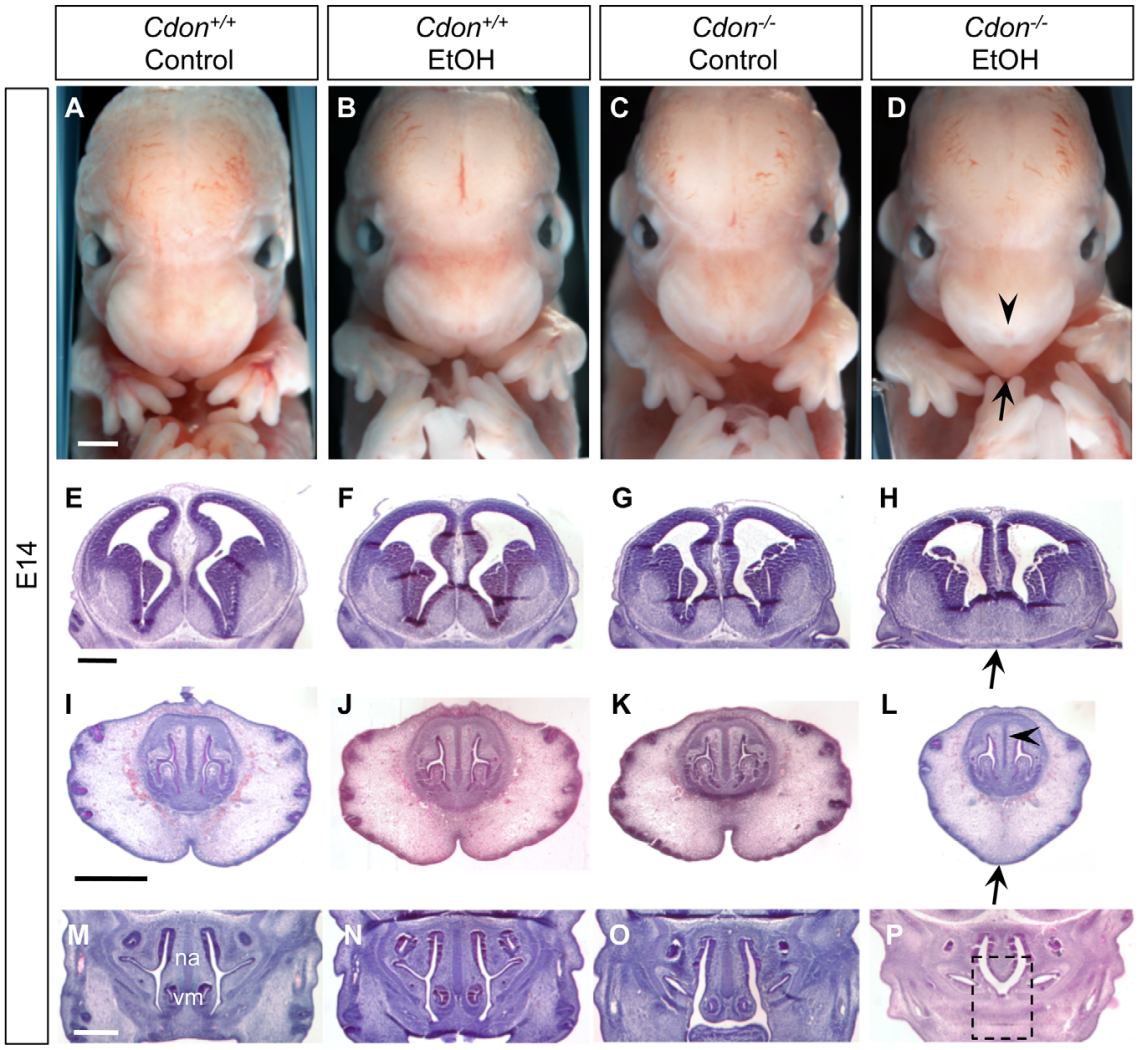
Synergy between loss of *Cdon* and fetal ethanol exposure produces HPE: E14.0. (A–D) Frontal views of E14.0 embryos. EtOH-treated *Cdon^−/−^* embryos (D) displayed strong facial features of HPE, including a single nostril (arrowhead) and smooth, pointed philtrum (arrow). (E–P) H&E-stained coronal sections of E14.0. (E–H) EtOH-treated *Cdon^−/−^* embryos had lobar HPE as indicated by continuity across the ventral midline (H, arrow). (I–L) Midfacial midline structures were also reduced in EtOH-treated *Cdon^−/−^* embryos, including the cartilage primordium of the nasal septum (L, arrowhead), philtrum and upper lip (L, arrow). (M–P) The vomeronasal organ (vm) and nasal septum (na) were strongly reduced in EtOH-treated *Cdon^−/−^* embryos (P, dashed box). Scale bars, 1 mm.

**Figure 3:**
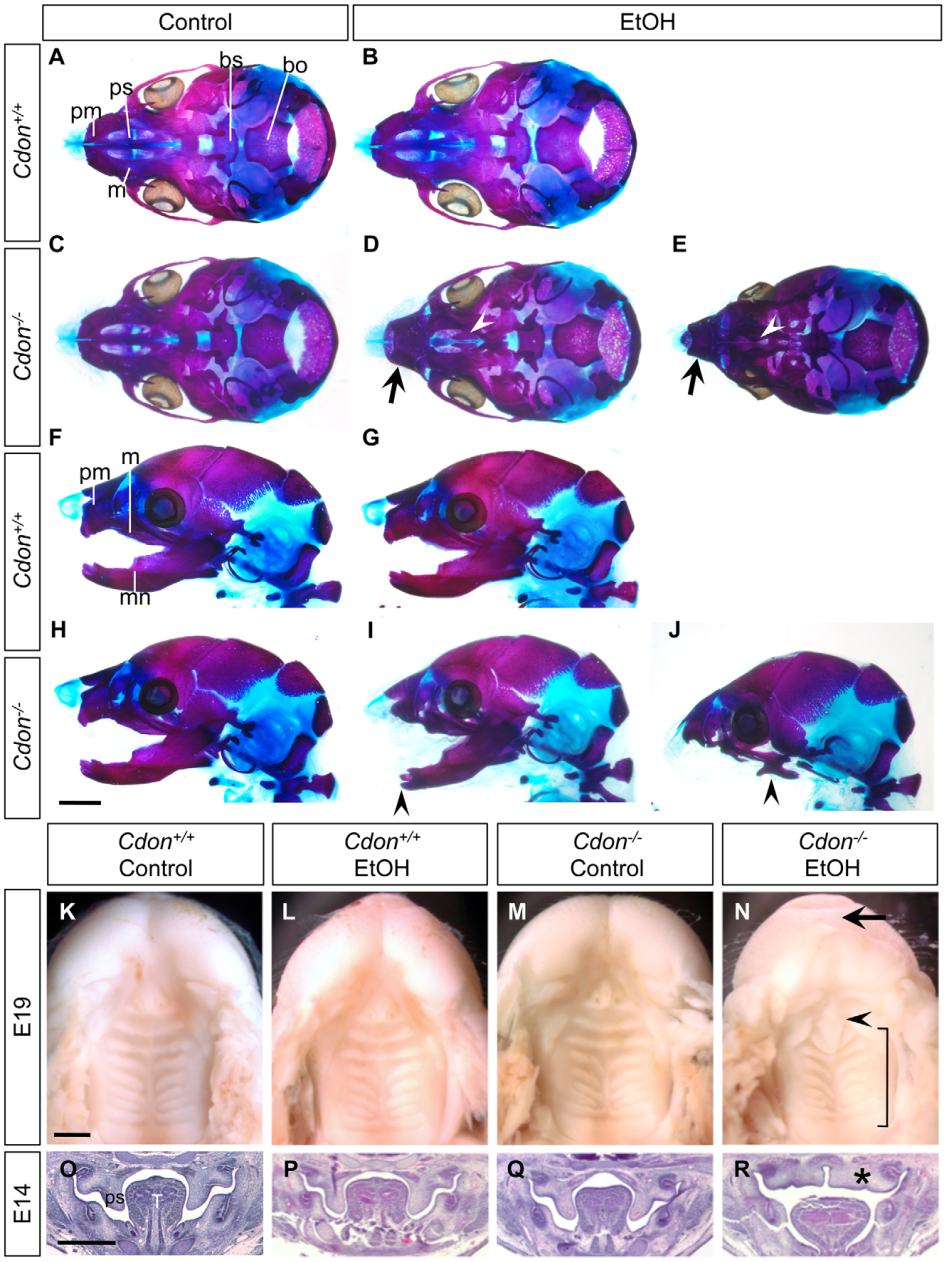
Cranial bone and palatogenesis defects in ethanol-treated *Cdon^−/−^* mice. Skeletal preparations of E19.0 mice of the indicated genotype and treatment were stained with alizarin red and alcian blue to identify bone and cartilage, respectively. (A–E) Ventral view of skulls, lower jaw removed. EtOH-treated *Cdon^−/−^* mice, but not other conditions, displayed shortened, fused premaxillary bones (black arrows) and underdeveloped maxillary shelves (white arrowheads). (F–J) Lateral views showing mandibular defects, including shortened, fused mandibular bones (I, arrowhead) or complete agnathia (J, arrowhead). pm, premaxillary bone; ps, palatal shelf; m, maxillary bone; bs, basisphenoid bone; bo, basooccipital bone; mn, mandibular bone. (K–N) Ventral views of E19.0 heads of the indicated genotype and treatment. The lower jaws were removed to visualize the palate. EtOH-treated *Cdon* mutants (N) had a smooth philtrum (arrow) and abnormal patterning of the secondary palate (bracket). The primary palate was present but misshapen (arrowhead). (O–R) H&E-stained coronal sections of E14.0 embryos. Defective outgrowth of palatal shelves (ps) in EtOH-treated *Cdon^−/−^* embryos is indicated by the asterisk. T, Tongue; Scale bars (A–J), 2 mm; (K–R), 1 mm.

We examined E10.0 embryos (30–34 somites) for defects in rostroventral midline formation by measuring the distance between the left and right nasal pits (Figure 1A–1E). Ethanol-treated *Cdon^−/−^* embryos had significantly reduced distance between the nasal pits, as compared to saline-treated *Cdon^−/−^* embryos and saline- or ethanol-treated *Cdon^+/+^* embryos, none of which were different from each other (Figure 1J, 1K). Furthermore, whole-mount and section analyses revealed that 13.5% (n = 111) of E10.0 ethanol-exposed *Cdon^−/−^* embryos displayed a severe HPE phenotype, including loss of telencephalic structure, failure to divide the eye field, and absence of Rathke's pouch (Figure 1A–1I, Table 1). This is a more pronounced phenotype than that seen even in *Cdon^−/−^* embryos on a sensitized genetic background [28]. These most severely affected embryos died *in utero* and at E11.0 were in the process of resorption; >70% (n = 37) of the remaining E14.0–E19.0 *Cdon^−/−^* embryos had phenotypes that ranged from lobar HPE (characterized by a partitioned forebrain with abnormal ventral midline continuity) with single nostril, deficient philtrum, diminished nasal septal cartilage, and rudimentary vomeronasal organs, to microform HPE (Figure 2, Table 1). We note that lobar HPE is a relatively subtle forebrain phenotype also seen in *Cdon;Boc* double mutants on this background [27], whereas microform HPE is restricted to the facial midline and is often found in *Cdon^−/−^* mutants of a mixed genetic background [30]. Additionally, some ethanol-treated E14.0 *Cdon^−/−^* mice displayed coloboma (data not shown), an eye phenotype sometimes associated with human HPE [33]. E19.0 cranial bone/cartilage preparations revealed that, whereas saline controls of any genotype and ethanol-treated *Cdon^+/+^* mice had normal cranial and palatal bone patterning, up to 71% (n = 14) of ethanol-treated *Cdon^−/−^* mice displayed underdeveloped maxillary shelves and a foreshortened and/or fused premaxillary bone, both HPE-associated midline defects [27], [30] (Figure 3A–3E, Table 1). Furthermore, four of five ethanol-treated *Cdon^−/−^* mice, but none of the other mice, had misshapen primary and secondary palates (Figure 3K–3N, Table 1). Consistent with these E19.0 palate defects, coronal sections of E14.0 embryos revealed defective outgrowth of palatal shelves in ethanol-treated *Cdo^−/−^* embryos (Figure 3O–3R). The mandible is generally spared in HPE but SHH signaling is required for mandibular development [34], and agnathia spectrum phenotypes (i.e., hypoplasia through complete loss of the mandible) have been reported in up to 10% of HPE patients [35]. Three of 14 ethanol-treated E19.0 *Cdon^−/−^* mice displayed agnathia spectrum defects, two with fused, hypoplastic mandibles and one with complete agnathia (Figure 3F–3J, Table 1). Therefore, the synergistic interaction of loss of *Cdon* and fetal ethanol exposure resulted in a wide spectrum of HPE defects at high penetrance and also produced anomalies more rarely associated with human HPE, at a similar low penetrance.

**Table 1:** Frequency of HPE spectrum defects in ethanol-treated 129S6.*Cdon^−/−^* mice at different stages of analysis.

Stage of analysis	Defect	Treatment	Genotype (# affected/total (%))	
			*Cdon^+/+^*	*Cdon^−/−^*
E10.0	Alobar HPE/severe forebrain and eye field defects	Saline	0/49	0/35
		Ethanol	0/115	15/111 (13.5%)*
E14.0	External HPE features	Saline	0/36	1/22 (4.5%)
		Ethanol	0/12	13/18 (72.2%)*
E14.0	Lobar HPE	Saline	0/3	0/4
		Ethanol	0/3	2/4 (50%)**
E14.0	Diminished nasal septal cartilage and vomeronasal organ	Saline	0/3	0/4
		Ethanol	0/3	4/4 (100%)*
E14.0	Defective palatogenesis	Saline	0/3	0/4
		Ethanol	0/3	2/4 (50%)**
E19.0	External midline defects	Saline	0/6	0/11
		Ethanol	0/6	14/19 (73.7%)*
E19.0	Deficient philtrum	Saline	0/6	0/11
		Ethanol	0/6	13/18 (72.2%)*
E19.0	Single nostril	Saline	0/6	0/11
		Ethanol	0/6	8/18 (44.4%)*
E19.0	Shortened premaxillary bone	Saline	0/6	0/11
		Ethanol	0/6	7/14 (50%)*
E19.0	Fused premaxillary bone	Saline	0/6	0/11
		Ethanol	0/6	10/14 (71.4%)*
E19.0	Underdeveloped maxillary shelves	Saline	0/6	0/11
		Ethanol	0/6	8/14 (57.1%)*
E19.0	Agnathia spectrum phenotypes^1^	Saline	0/6	0/11
		Ethanol	0/6	3/14 (21.4%)*
E19.0	Abnormal patterning of secondary palate	Saline	0/4	0/4
		Ethanol	0/4	4/5 (80%)*

* p<0.05, when comparing ethanol-treated *Cdon^−/−^* embryos with saline and ethanol-treated *Cdon^+/+^* embryos and saline-treated *Cdon^−/−^* embryos.

** p>0.05, due to the relatively low n (4) for this analysis and the partial penetrance of the phenotype. However, in our experience, lobar HPE and defective palatogenesis are not observed in either *Cdon^+/+^* or *Cdon^−/−^* embryos on the 129S6 background [27], [28].

1 Agnathia spectrum phenotypes include complete lack of mandible (1/14) and fused, shortened mandibular bone (2/14).

*Cdon^−/−^* mice display HPE with strain-dependent severity. Mice lacking the *Cdon* paralog *Boc* do not have HPE, regardless of genetic background, but removal of *Boc* from *Cdon^−/−^* mice enhances their HPE phenotype [27]. We therefore tested whether *Boc^−/−^* embryos are also sensitized to ethanol-induced HPE. In contrast to *Cdon^−/−^* embryos, *Boc^−/−^* embryos exposed in utero to ethanol under the same protocol did not display detectable HPE or facial midline phenotypes at E14.0 (Figure S2).

### Inhibition of SHH expression and signaling in the ventral forebrain of ethanol-treated *Cdon^−/−^* mice

**Figure 4:**
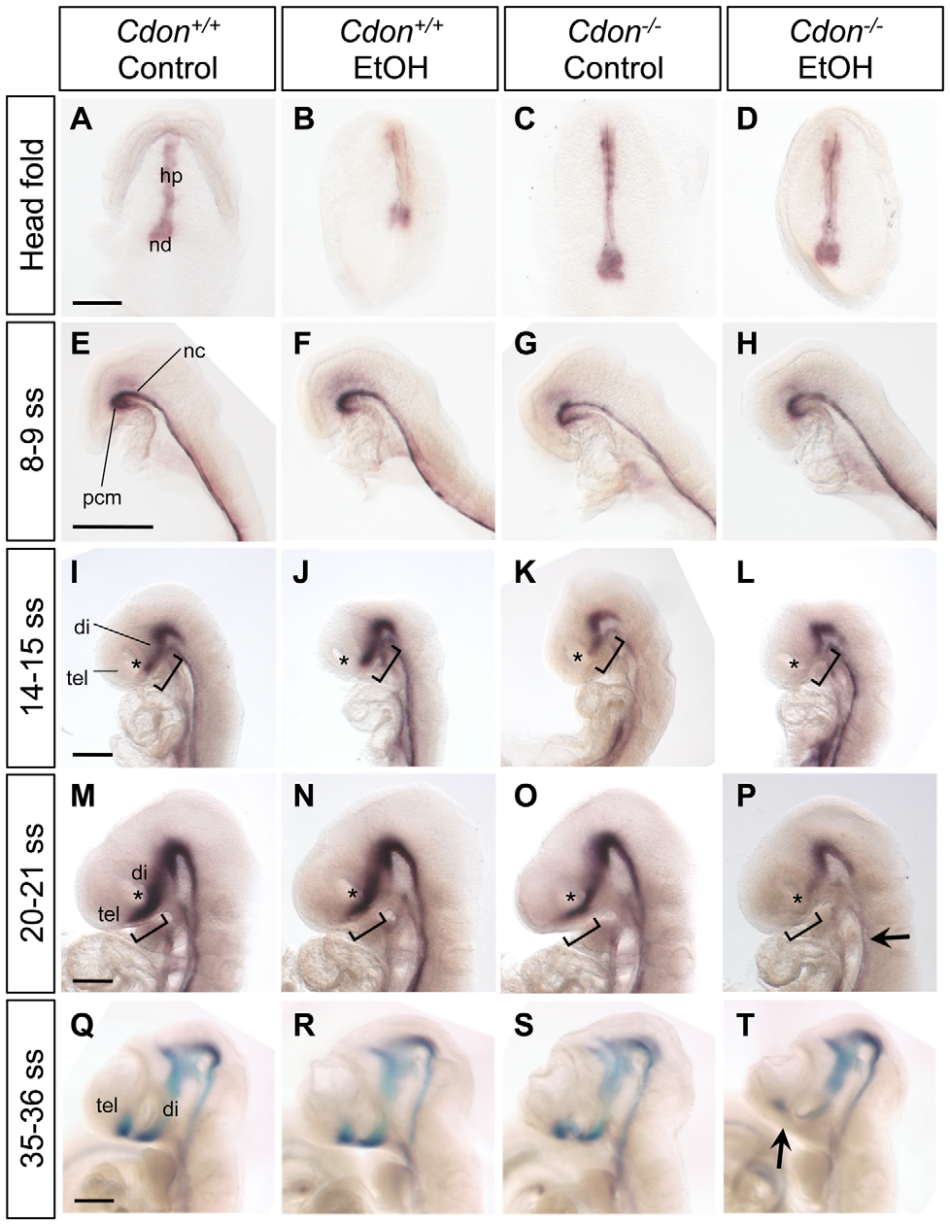
Defective expression of *Shh* in the ventral forebrains of ethanol-treated *Cdon^−/−^* embryos. Whole mount in situ hybridization analysis of *Shh* expression in embryos of the indicated genotype and treatment at: (A–D) the headfold stage (ventral views); (E–H) 8–9 somite stage (ss) embryos; (I–L) 14–15 ss; (M–P) 20–21 ss; and (Q–T) 35–36 ss (E–T, lateral views). *Shh* expression in the ventral diencephalon and telencephalon is diminished specifically in EtOH-treated *Cdon* mutants from 14–15 somites and onward. Asterisks (I–P) denote the developing eye, which serves as a landmark in the rostrocaudal axis of the developing head; brackets (I–P) denote the expression domain in control *Cdon^+/+^* embryos of the indicated ss and highlight the deficit in *Shh* expression in the most rostral portion of the *Shh* expression domain, at that stage. The arrow in (P) indicates reduced *Shh* expression in the floorplate; the arrow in (T) indicates reduced *Shh* expression in the telencephalon. hp, head process; nd, node; pcm, prechordal mesendoderm; nc, notochord; tel, telencephalon; di, diencephalon. Scale bars, 250 µm.

SHH produced by the prechordal mesendoderm (PCM) is required for initiating development of the midline of the forebrain and midface [36]. PCM-derived SHH induces expression of *Shh* itself and SHH pathway target genes in the ventral midline of the developing diencephalon and, subsequently, telencephalon [37], [38]. Expression of *Shh* in the ventral diencephalon also requires *Six3*, an HPE gene that encodes a homeodomain transcription factor [39], [40]. We used whole-mount *in situ* methods to assess *Shh* and *Six3* expression, as well as apoptosis and cell proliferation, at 24–36 hr after the initial *in utero* ethanol treatment of embryos. Ethanol treatment had no effect on *Shh* expression in the axial mesoderm at the headfold (presomite) stage, or in the PCM and notochord at the 8–9 somite stage (Figure 4A–4H; Table 2). Furthermore, *Six3* was expressed normally in the ventral forebrain of ethanol-treated *Cdon^−/−^* embryos of 8–9 somites, just prior to *Shh* induction in that structure (Figure S3; Table 3). Ethanol did not induce apoptosis in the midline of 4–6 somite embryos, although the number of TUNEL^+^ cells in the lateral regions of the anterior neural plate was increased by ethanol in a manner independent of *Cdon* genotype (Figure S4). Cell proliferation, as assessed by immunostaining for phospho-histone H3, was unaffected at the 4–6 somite stage by either ethanol treatment or *Cdon* genotype (Figure S4). Taken together, *Cdon^−/−^* embryos exposed to ethanol did not display obvious alterations in several aspects of rostroventral midline development 24–36 hr after ethanol treatment.

**Table 2:** Defects in *Shh* expression in ethanol-treated 129S6.*Cdon^−/−^* mice at different stages of analysis.

Stage of analysis	Structure	Treatment	Genotype (# affected/total)	
			*Cdon^+/+^*	*Cdon^−/−^*
Presomite headfold (E7.5–8.0)	Axial mesoderm	Saline	0/7	0/5
		Ethanol	0/6	0/5
8–9 somites (E8.25)	Prechordal mesendoderm and notochord	Saline	0/4	0/4
		Ethanol	0/4	0/6
14–15 somites (E9.0)	Ventral forebrain	Saline	0/3	1/3*
		Ethanol	0/3	4/4
20–21 somites (E9.25)	Ventral telencephalon	Saline	0/3	2/4*
		Ethanol	0/3	4/4
35–36 somites (E10.25)	Ventral telencephalon	Saline	0/5	0/2
		Ethanol	0/5	3/5

* Small decreases in signal strength, but not changes in the expression pattern, were seen as previously reported [27].

**Table 3:** Defects in gene expression in ethanol-treated 129S6.*Cdon^−/−^* mice at different stages of analysis.

Stage of analysis	Gene	Treatment	Genotype (# affected/total)	
			*Cdon^+/+^*	*Cdon^−/−^*
Late streak (E7.5)	*Foxa2*	Saline	0/4	0/3
		Ethanol	0/5	3/5
Late streak (E7.5)	*Gsc*	Saline	0/4	0/4
		Ethanol	0/3	2/4
Early allantoic bud (E8.0)	*Cer1*	Saline	0/4	0/4
		Ethanol	0/5	1/6
8–9 somites (E8.25)	*Six3*	Saline	0/2	0/2
		Ethanol	0/2	0/3
35–36 somites (E10.25)	*Gli1*	Saline	0/2	0/2
		Ethanol	0/2	3/5
35–36 somites (E10.25)	*Ptch1*	Saline	0/2	0/2
		Ethanol	0/2	1/5*
35–36 somites (E10.25)	*Nkx2.1*	Saline	0/2	0/2
		Ethanol	0/2	3/5
35–36 somites (E10.25)	*Fgf8*	Saline	0/2	0/2
		Ethanol	0/5	2/5
35–36 somites (E10.25)	*Msx1*	Saline	0/2	0/2
		Ethanol	0/2	0/5
35–36 somites (E10.25)	*Msx2*	Saline	0/2	0/2

**Figure 5:**
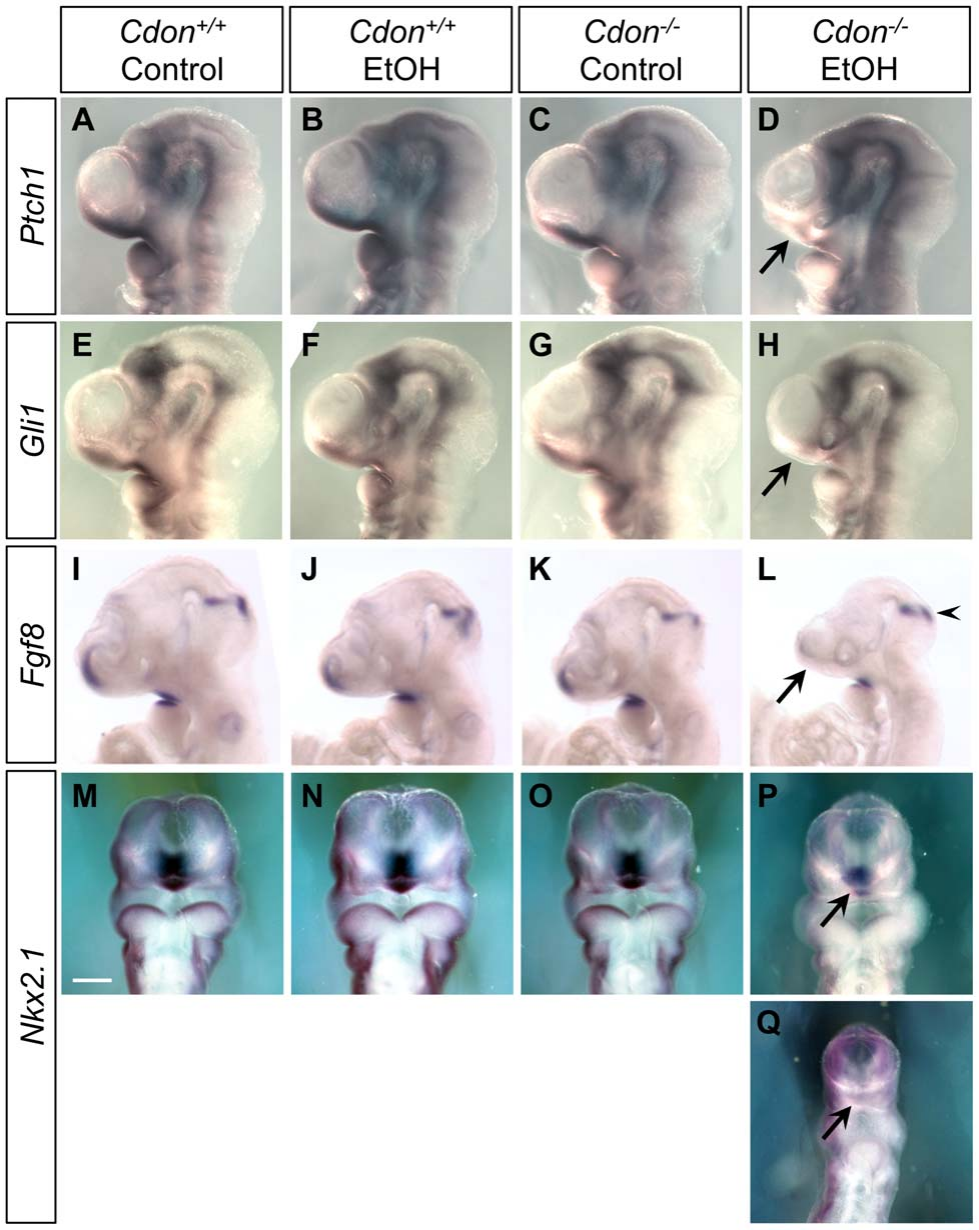
Defective expression of SHH pathway target genes in the ventral forebrains of ethanol-treated *Cdon^−/−^* embryos. Whole mount in situ hybridization analyses of *Ptch1* (A–D), *Gli1* (E–H), *Fgf8* (I–L) and *Nkx2.1* (M–Q) expression in E10.0 (30–36 somites) embryos of the indicated genotype and treatment (A–L, lateral views; M–Q, ventral views). *Ptch1* and *Gli1* expression in the ventral telencephalon was decreased in EtOH-treated *Cdon* mutants (*D* and *H*, arrows). *Fgf8* expression in the commissural plate but not at the midbrain-hindbrain boundary, was diminished in EtOH-treated *Cdon* mutants (L, arrow and arrowhead, respectively). Expression of the ventral forebrain marker *Nkx2.1* was diminished by ethanol treatment of *Cdo* mutants (P and Q, arrows; P and Q show moderately and severely affected embryos, respectively). Scale bar, 250 µm.

* One of 5 embryos showed a strong loss of *Ptch1* expression in the ventral forebrain (shown in Figure 5D); the remaining four showed a pattern similar to controls but with reduced signal.

We next examined expression of *Shh* and SHH target genes in the developing forebrain at stages just subsequent to this period. *Shh* expression in the diencephalon was initiated normally at the 14–15 somite stage in ethanol-treated *Cdon^+/+^* embryos and in saline-treated *Cdon^−/−^* embryos (Figure 4I–4K). In contrast, *Shh* expression in the rostral diencephalon was strongly reduced in ethanol-exposed *Cdon^−/−^* embryos at this stage (Figure 4L; Table 3). This pattern of diminished *Shh* expression in ethanol-treated *Cdon* mutants was also observed at later stages when, in control embryos, the expression zone expanded anteriorly in the diencephalon (20–21 somites) (Figure 4M–4P; Table 3), and when *Shh* had been induced in the ventral telencephalon (35–36 somites) (Figure 4Q–4T; Table 3). Consistent with the reduction in *Shh* expression in the ventral forebrain of ethanol-treated *Cdon^−/−^* embryos, expression of the direct SHH target genes *Ptch1*, *Gli1* and *Nkx2.1* was reduced specifically in the ventral forebrain of these embryos at the 35–36 somite stage (Figure 5A–5H, 5M–5Q). Reduction in SHH target gene expression was seen with partial penetrance (Table 2), similar to the partial penetrance and range of HPE phenotypes produced by this protocol (Table 1). SHH is required for maintenance of *Fgf8* expression in the commissural plate of the rostral telencephalon but not expression at the midbrain-hindbrain boundary [41], and *Fgf8* expression in the former, but not the latter, structure was diminished in ethanol-treated *Cdon* mutants at this stage (Figure 5I–5L; Table 2). As a control for specificity of inhibition of SHH-dependent gene expression, we assessed expression of *Msx1* and *Msx2*, which are markers of the migrating neural crest cells that contribute to craniofacial structures but are not targets of SHH signaling [34], [42]. The expression patterns of *Msx1* and *Msx2* were not affected in ethanol-treated *Cdon^−/−^* embryos (Figure S5; Table 2). We conclude that synergistic interaction between mutation of *Cdon^−/−^* and ethanol exposure during early development results in delayed and diminished induction of *Shh* expression specifically in the ventral forebrain, leading to a failure to pattern the rostroventral midline and, consequently, to HPE.

To apply a more quantitative approach to these results, RNA was extracted from dissected forebrains of E10.0 embryos, and quantitative RT-PCR (qRT-PCR) was performed. Although there was some variability, likely due to the partial penetrance observed at the level of in situ hybridization (Table 1 and Table 2), mRNA levels of *Shh*, *Nkx2.1*, *Ptch1*, *Fgf8* and *Gli1* were each reduced in the ethanol-treated *Cdon^−/−^* embryonic heads relative to controls (Figure S6). Moreover, it is likely that the qRT-PCR results underestimate the reduction in expression of these genes in the most affected region of ethanol-treated *Cdon^−/−^* embryos (the rostroventral midline) because more caudal and lateral forebrain structures, where changes in expression were not obvious, were by necessity included in the dissected tissue.

SHH signaling is required for dorsoventral patterning of the neural tube [43], and *Cdon^−/−^* embryos on a mixed 129S6×C57BL/6NTac background show a reduction in FOXA2^+^ floor plate cells [25]. Additionally, *Shh* levels were reduced in the floor plate of ethanol-treated *Cdon^−/−^* embryos at the 20–21 somite stage (Figure 4M–4P). We therefore tested whether ethanol-exposed *Cdon^−/−^* embryos had defective neural tube patterning. Sections of saline- and ethanol-treated control and *Cdon^−/−^* embryos were analyzed by immunofluorescence at E10.0 for FOXA2, NKX2.2, NKX6.1, PAX6 and PAX7, markers whose respective expression zones span the entire dorsoventral axis of the developing neural tube. No differences in the expression patterns of any of these factors were seen between any of the four conditions (Figure S7). Furthermore, the neural tube of ethanol-exposed *Cdon^−/−^* embryos had a normal morphology. It is likely that the reduction in floor plate *Shh* expression in these embryos does not have an overt effect on further neural tube patterning because notochord-derived SHH was not perturbed and is largely sufficient for this process [43]. Therefore, the synergistic effect of loss of *Cdon* and in utero ethanol exposure was restricted to the most rostral portion of the ventral midline, resulting in HPE spectrum defects, but not obvious neural tube defects.

### Diminished expression of *Gsc* and *Foxa2* in early ethanol-treated *Cdon^−/−^* embryos

**Figure 6:**
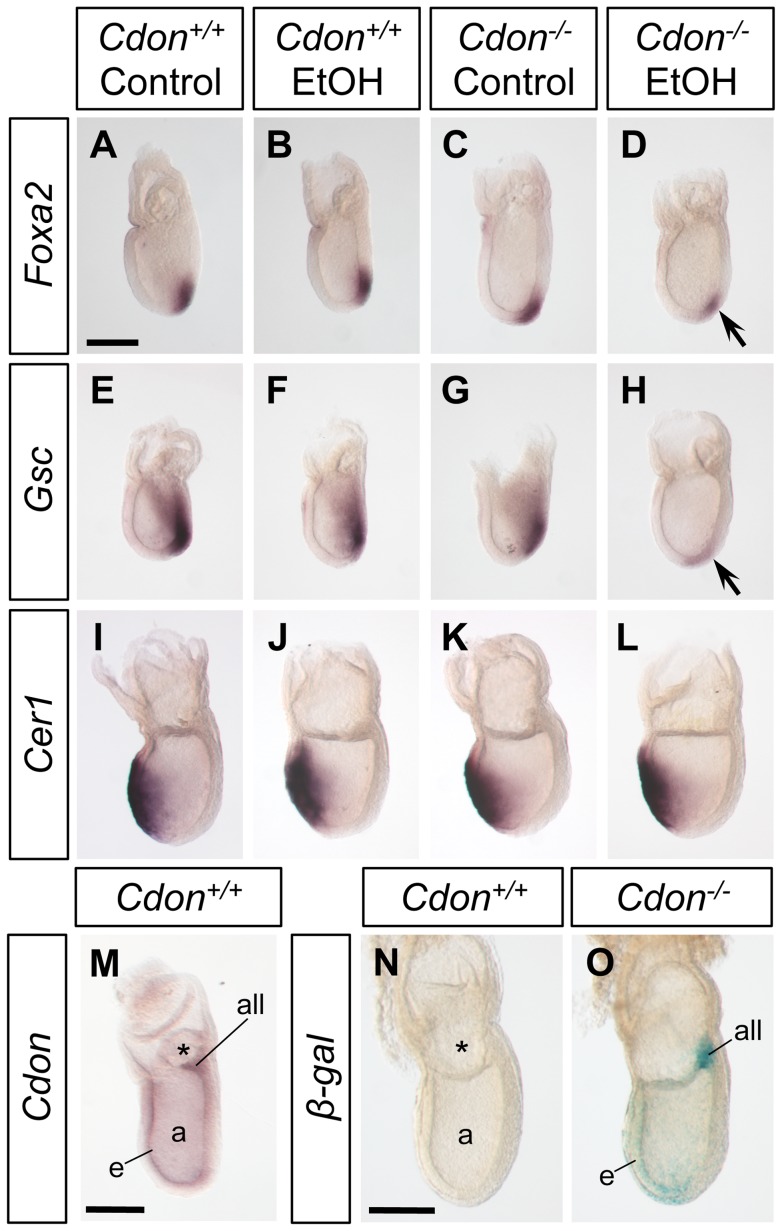
Defective expression of *Foxa2* and *Gsc* at the late streak stage of ethanol-treated *Cdon^−/−^* embryos. Whole mount in situ hybridization analyses of *Foxa2* (A–D), *Gsc* (E–H) and *Cer1* (I–L) in embryos of the indicated genotype and treatment harvested at E7.25. *Foxa2* and *Gsc* expression was analyzed at the late streak stage and was decreased in EtOH-treated *Cdon* mutants (D and H, arrows). *Cer1* was analyzed at the early bud stage and was unaffected by loss of *Cdon* or EtOH treatment. (M) Whole mount in situ hybridization analysis of *Cdon* expression in a wild-type E7.25 embryo at the late streak stage. *Cdon* expression is observed in the ectoderm, mesoderm and allantoic bud. *Cdon* expression was also monitored via ß-galactosidase (ß-gal) activity derived from a *LacZ* reporter knocked into the *Cdon* locus in mutant E7.5 embryos (O) at the early bud stage; a wild-type embryo stained for ß-gal activity is shown as a negative control (N). *Cdon* expression is seen mainly in ectoderm, mesoderm and allantois. a, amniotic cavity; all, allantois; e, ectoderm; m, mesoderm; *, exocoelom. Scale bars, 200 µm.

The timing and location of defects in *Shh* expression and signaling shown in Figure 3 and Figure 4 are consistent with the HPE phenotypes seen in the majority of ethanol-treated *Cdon^−/−^* embryos (Figure 2). However, the alobar HPE found in 13.5% of E10.0 embryos is less easily explained by such alterations in SHH pathway activity. Furthermore, it must be presumed that ethanol exerts its effects during the relatively brief window in which *Cdon^−/−^* embryos are exposed (peak levels occur at approximately E7.25; Figure S1), even if defects in midline development occur subsequently. Patterning events that result in the formation of the PCM occur during the period of ethanol exposure; this process is regulated by the NODAL signaling pathway, and mutations in NODAL pathway components are observed in human HPE [6], [44]. *Foxa2* and *Gsc* (*Goosecoid*) are two markers of the anterior primitive streak, from which the PCM is derived, and they function cooperatively to specify anterior mesendoderm [45]–[47]. We found that some ethanol-treated *Cdon^−/−^* embryos at the late streak stage (isolated at E7.25–E7.5) displayed substantially reduced expression of *Foxa2* (3 of 5 embryos) and *Gsc* (2 of 4 embryos) as compared to saline-treated *Cdon^+/+^* controls (Figure 6A–6H; Table 3). This decrease in *Foxa2* and *Gsc* expression was not due simply to the brief delay in development seen in all ethanol-treated embryos (see above and Table S2) because ethanol-exposed *Cdon^+/+^* embryos did not show obvious defects in *Foxa2* or *Gsc* expression (Figure 6A–6H; Table 3). Saline-treated *Cdon^−/−^* embryos also resembled controls (Figure 6A–6H; Table 3). Therefore, these defects arose as a consequence of an interaction between ethanol exposure and loss of *Cdon*. Similar effects on *Foxa2* and *Gsc* expression are seen in embryos with defects in NODAL signaling [48]–[50]. Another derivative of the anterior primitive streak is the anterior definitive endoderm (ADE), and embryos with defective NODAL signaling display decreased expression of the ADE marker *Cer1* (*Cerberus 1*) at the late streak and early allantoic bud stages [49], [50]. In contrast to *Foxa2* and *Gsc* expression, *Cer1* expression at the early bud stage was not obviously altered by ethanol treatment in 5 of 6 *Cdon^−/−^* embryos (Figure 6I–6L; Table 3).

The synergy between ethanol exposure and loss of CDON is therefore detectable at a developmental stage prior to SHH function [51]. Consistent with these early effects of ethanol treatment in *Cdon^−/−^* embryos, we find that *Cdon* is expressed at the late-streak and early bud stages in the ectoderm, mesoderm and allantoic bud as assessed by in situ hybridization and ß-galactosidase activity derived from a *LacZ* reporter knocked into the *Cdon* locus (Figure 6M–6O). To assess whether treatment with ethanol at this early stage was critical to production of HPE spectrum phenotypes, pregnant females were administered ethanol at E8.0, rather than E7.0, and embryos collected at E14.0 for analysis. In contrast to the external features of HPE seen in 13 out 18 *Cdon^−/−^* embryos treated at E7.0 and analyzed at E14.0 (Table 1), none of 13 *Cdon^−/−^* embryos treated at E8.0 displayed such phenotypes when analyzed at this stage (Figure S8).

## Discussion

Risk factors for HPE are heterogeneous, and the spectrum of defects in humans is characterized by highly variable phenotypic severity, even within pedigrees [1], [12]. It has been proposed that these observations are best explained by multiple-hit models, in which the penetrance and expressivity of a heterozygous loss-of-function mutation may be enhanced by a second mutation or the presence of cooperating, but otherwise silent, modifier genes that may be present at much higher frequency in the human population [1], [5], [14], [44]. Recent, detailed analysis of a large HPE cohort offers support for the occurrence of multiple genetic events in specific individuals but such cases are a small percentage of the total [13]. Non-genetic risk factors are also implicated in human HPE, and certain teratogens can cause HPE in animals [15], [16], [20]. It seems extremely likely, therefore, that gene-environment interactions may be involved in the etiology of HPE as an alternative multiple-hit model to purely genetic multiple-hit models. There is, however, little or no evidence for this contention. We report here, for the first time, an animal model in which there is dramatic synergy between mutation of a bona fide HPE gene, *Cdon*, and a suspected HPE teratogen, ethanol.

### CDON function in midline patterning and HPE

CDON functions as a co-receptor in the SHH pathway, binding both to Hedgehog ligand and to the primary receptor, PTCH1 [9], [52]. Loss-of-function missense mutations in *CDON* identified in human HPE cases result in variant proteins that do not support ligand-dependent signaling and display defective interactions with PTCH1 [9]. Previous studies with *Cdon^−/−^* embryos on sensitive and resistant genetic backgrounds (C57BL/6NTac and 129S6, respectively) argued that loss of CDON results in disruption of SHH signaling in and/or from the PCM, leading to defective induction of *Shh* and SHH target gene expression in the ventral forebrain, with consequent effects on forebrain and facial midline patterning [27], [28], [30]. The precise timing and location of the first observable defect in *Shh* expression in ethanol-treated 129S6.*Cdon^−/−^* embryos (between the 8–9 and 14–15 somite stages in the rostroventral diencephalon), as well as the severity of the forebrain and facial midline phenotypes in the majority of such embryos (lobar HPE with strong midfacial anomalies), is similarly consistent with a defect in SHH signaling from the PCM to the presumptive ventral forebrain and/or responsiveness of the latter to PCM-derived inductive signals. However, 13.5% of ethanol-treated *Cdon^−/−^* embryos had severe HPE at E10.0 (and were being resorbed by E11.0), a stronger phenotype than that anticipated by the above mechanism. Furthermore, ethanol treatment at E8.0, rather than the standard E7.0, did not produce HPE in *Cdon^−/−^* embryos. The PCM arises from the anterior primitive streak. Development of the anterior primitive streak occurs during the time of embryonic ethanol exposure with E7.0 administration, and defects in this developmental process are associated with HPE [6], [44]. We therefore analyzed expression of *Foxa2* and *Gsc*, two genes that mark the anterior streak and function to pattern the PCM [45]–[47]. Fifty-to-sixty percent of ethanol-treated *Cdon^−/−^* embryos displayed substantially reduced expression of these genes. Although ethanol caused a transient, two- to four-hour delay in development that was independent of embryo genotype, only *Cdon^−/−^* embryos showed decreased expression of *Foxa2* and *Gsc* in response to ethanol.

*Nodal^+/−^;Gdf1^−/−^* mice and *Nodal^+/−^;Chrd^−/−^* mice, which have defective NODAL pathway signaling (and, at least in the case of *Nodal^+/−^;Chrd^−/−^* mice, overactive BMP signaling) show a similar diminution of *Foxa2* and *Gsc* expression and develop HPE [48]–[50]. This stage of development is prior to expression of *Shh*, but we report here that *Cdon* is expressed at the late streak and early bud stages. These results suggest that CDON plays an earlier role in development than its known role as a SHH co-receptor. CDON is a multifunctional co-receptor, and promotes signaling in a Hedgehog-independent manner when associated with various other cell adhesion molecules and signaling receptors [53], [54]. Perhaps CDON is also able to function with NODAL, BMP or other ligands or their antagonists; it should be noted, however, that embryos with defective NODAL pathway signaling also display reduction in expression of the ADE marker, *Cer1* [49], [50], and this was not observed in ethanol-treated *Cdon^−/−^* embryos. Although the mechanism whereby CDON exerts effects in primitive streak stage embryos is not clear, the need for ethanol exposure to reveal this role suggests that, in the absence of additional insults, it is subtle or redundant with other factors. A model consistent with all these data is that CDON can function at multiple points in rostroventral midline patterning, one of which is via promotion of SHH signaling. In 129S6 mice, ethanol initiates defects in midline patterning specifically in genetically sensitized (i.e., *Cdon^−/−^*) embryos, with the variable severity of the HPE phenotype – ranging from severe HPE to no overt effect beyond that associated with loss of *Cdon* alone on this background – arising stochastically. It will be interesting to test in the future whether mice carrying mutations specific for the SHH pathway (e.g., *Shh^+/−^* mice) are sensitized to ethanol treatment; similarly, animals potentially sensitized by heterozygosity for NODAL pathway components could also be investigated.

### Gene–environment interaction and production of a broad spectrum of HPE phenotypes

A previous study on chick embryos treated at various developmental stages with the Hedgehog pathway inhibitor cyclopamine concluded that a phenotypic HPE spectrum could be produced by varying the timing of SHH pathway blockade; i.e., early inhibition led specifically to severe phenotypes, while later time points of inhibition led to progressively less severe defects [37]. Our findings indicate that brief exposure to a teratogen early in development can produce a similar broad range of phenotypes in a genetically susceptible host: transient ethanol exposure during gastrulation of 129S6.*Cdon^−/−^* mice, produced not only alobar HPE, which is associated with early patterning defects, and but also milder forms of HPE with HPE-related phenotypes that are associated with much later patterning defects (e.g., in palatogenesis).

Fetal alcohol exposure has been linked to HPE and alterations in SHH pathway activity in other animal models, including mice and zebrafish [17]–[22], [55], but a specific genetic interaction between ethanol and the SHH pathway has not been reported. Most mouse strains, including 129S6, are resistant to ethanol, and ethanol induces HPE only with low penetrance even in the most widely studied strain, C57BL/6J [17]–[19]. In zebrafish, ethanol produces cyclopia but also severe defects along the entire anterior-posterior axis [21]. In contrast, the 129S6.*Cdon^−/−^* plus in utero ethanol-treatment model is notable for its specificity, including: 1) timing of exposure (administration of ethanol at E7.0 but not E8.0 was effective); 2) structures affected (the ventral forebrain and craniofacial midline displayed defects but the neural tube did not); and 3) mutation of a bona fide HPE gene, *Cdon*, but not an HPE modifier gene, *Boc*, synergized with ethanol. Therefore, this model incorporates major known and predicted features of human HPE, including: 1) a multifactorial etiology that reveals gene-environment interactions in the specific inhibition of SHH pathway activity in the rostroventral midline; and 2) a broad spectrum of HPE phenotypes, including low penetrance phenotypes such as agnathia.

**Figure 7:**
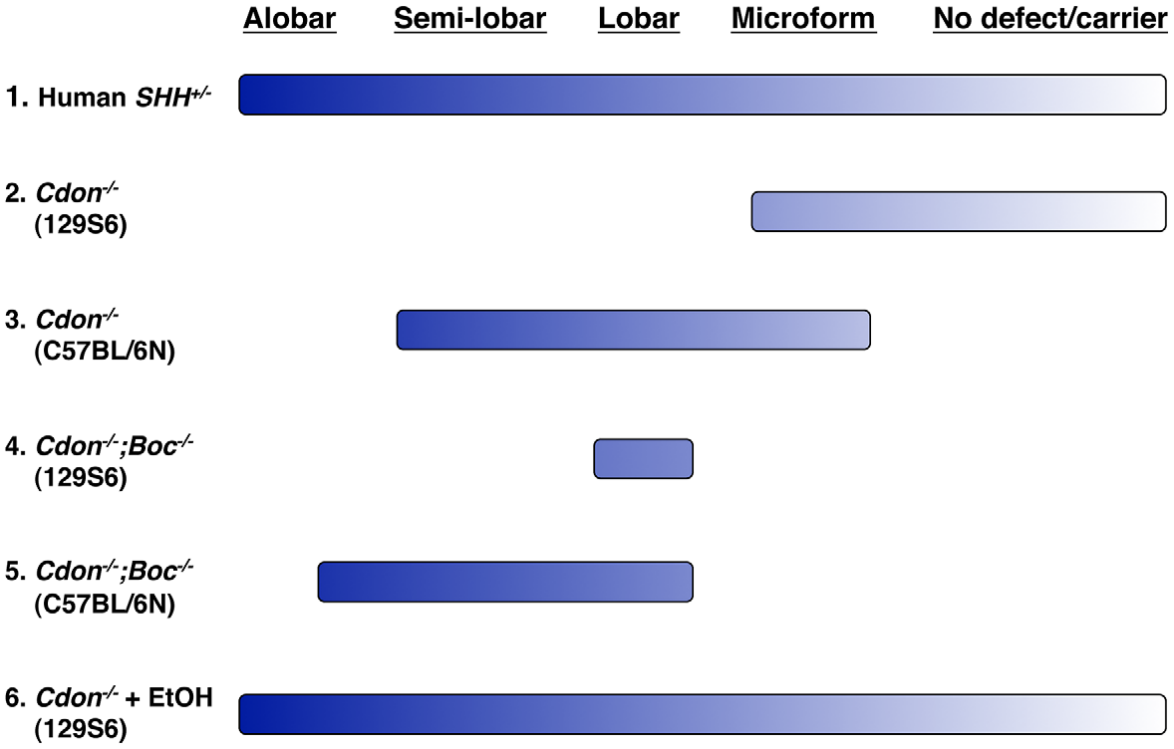
Spectrum of HPE phenotypes in various mouse models. The HPE spectrum of phenotypes is displayed across the top and ranges from alobar HPE in the most severe cases to no overt defect in carriers of HPE-associated mutations, such as heterozygosity for *SHH* (line 1). The respective ranges of HPE phenotypes seen in mice with mutations in *Cdon* plus or minus mutations in *Boc* on resistant (129S6) and sensitive (C57BL/6N) backgrounds are indicated by lines 2–4. The breadth of the lines represents the full range of phenotypes seen in the various lines of mice. Note that no combination of mutations and background results in a complete phenotypic spectrum. In contrast, ethanol-treated 129S6.*Cdon^−/−^* mice display a nearly full spectrum of phenotypes, similar to that seen in human HPE (line 5).

Genetic removal of *Cdon* plus or minus removal of the paralogous gene *Boc* on two different genetic backgrounds results in animals that display distinct windows within the range of HPE phenotypes observed in human cases (e.g., in individuals heterozygous for loss-of-function *SHH* mutations) (Figure 7). However, no combination of gene loss and strain background resulted in as wide a spectrum of HPE phenotypes as that seen in the human population, even within pedigrees. In contrast, ethanol-treated *Cdon^−/−^* mice display a nearly complete HPE spectrum, including low penetrance phenotypes (Figure 7).

These findings suggest that interaction between genetic and teratogenic factors may well underlie many HPE cases and have considerable public health and clinical implications. Approximately 12% of pregnant women use alcohol and 2% do so heavily (i.e., “binge” drinking) [56]. Furthermore, the time of exposure in this model [31] is equivalent to the third week of human gestation, a time when many women are unaware they are pregnant. Epidemiological evidence for ethanol as an HPE risk factor is ambiguous [15], [16]. The results presented here argue that ethanol is indeed a risk factor for HPE, but genetically predisposed individuals, such as those with SHH pathway mutations, may be particularly susceptible. Because 129S6.*Cdon^−/−^* mice are capable of identifying both genetic (e.g., *Boc*) and non-genetic (e.g. ethanol) risk factors for HPE that are insufficient to produce HPE alone (i.e., silent cofactors) ([25], [27]; this study), we propose that they serve as a potential animal model for the assessment and identification of plausible risk factors for HPE.

## Materials and Methods

### Ethics statement

All animal work was approved by the Institutional Animal Care and Use Committee (IACUC). Our animal facility is accredited by the Association for Assessment and Accreditation of Laboratory Animal Care International (AAALAC).

### Mice

Two to three month-old *Cdon^+/tm1Rsk^* (*Cdon^+/−^*) mice on a 129S6/SvEvTac (129S6) background [28], [30] were mated for one hour in the dark and plugged females were collected. The time of the plug was designated as embryonic day (E) 0.0. Ethanol administration was performed as described by Sulik et al. and Webster et al. [31], [32] with slight modification. In initial experiments, pregnant female mice were injected intraperitoneally twice with 15 µl per gm body weight of a solution of 25% ethanol in saline (2.9 gm/kg), at E7.0 and 4 hr later. Saline injections were used as a control. Offspring were examined at E14.0, and 22.2% of ethanol-treated *Cdon^−/−^* embryos (but not *Cdon^+/+^* or *Cdon^+/−^* embryos) displayed external signs of HPE (n = 56). Injected females were also assessed for blood alcohol concentration over 10 hr after the first injection with the Pointe Scientific Alcohol Reagent Set (A7504) (Figure S1). As previously reported [32], blood alcohol concentrations peaked 1 hr after the second injection, but the concentration achieved in 129S6 mice was only ∼60% that described for C57BL/6J mice, the most widely-used strain. We therefore increased the dose by 20% (15 µl per gm body weight of a solution of 30% ethanol in saline; 3.48 gm/kg). The blood alcohol levels achieved were very similar to those reported previously with the 2.9 gm/kg dose in C57BL/6J mice [32], and >70% of E14.0 *Cdon^−/−^* embryos (but not *Cdon^+/+^* or *Cdon^+/−^* embryos) under this protocol displayed external signs of HPE (Table S1). This protocol was used in the studies reported here unless noted otherwise. In some experiments 129S6.*Boc^+/−^* mice [27] were used in place of *Cdon^+/−^* mice and in others, plugged *Cdon^+/−^* females received ethanol at E8.0 rather than E7.0.

129S6 mice are resistant to ethanol teratogenicity ([18]; this study), and we note that these concentrations are above those generally achieved clinically in humans. Among more than 150 pregnant females treated with ethanol, ∼40% lost their litters in less than 24 hr but none died of acute toxicity. Results reported are from litters that survived this initial period, after which resorptions were unusual (Table S1). For all analyses, embryos were collected at the embryonic day indicated in the text. At presomitic stages, embryos were further staged by morphology and in situ hybridization. For E8.0 and later, embryos of equivalent somite number were compared. Although ethanol-treated embryos had, on average, between one and two fewer somite pairs than saline-treated controls regardless of genotype (see Results), analysis of multiple embryos that differed by one to two somites at the various stages shown in Figure 4 demonstrated that this transient delay was not the cause of the altered gene expression patterns seen in ethanol-treated *Cdon^−/−^* embryos.

### Histology and immunofluorescence

Embryos were dissected out and fixed overnight in 4% paraformaldehyde in PBS. Embryos were then dehydrated through a graded ethanol series, embedded in paraffin and sectioned at 8 µm. Hematoxylin and eosin (H&E) staining was performed as described [27]. Slides were then dehydrated through graded ethanol and xylene and mounted with Permount (Fisher Scientific).

Immunofluorescence analysis of developing neural tubes was performed on frozen sections as described [57], and images were taken on a Zeiss Axioplan 2 microscope. The number of cells expressing a particular marker, or the relative size of marker expression domains, was measured with ImageJ software. The antibodies used were: mouse anti-FOXA2 (1∶20; DSHB), mouse anti-NKX2.2 (undiluted; DSHB), mouse anti NKX6.1 (1∶20; DSHB), mouse anti-PAX6 (1∶20; DSHB), mouse anti-PAX7 (1∶20; DSHB), and Alexa 488 anti-mouse IgG (1∶500; Invitrogen).

### Whole-mount in situ hybridization, TUNEL assay, immunohistochemistry, and *ß*-galactosidase staining

For whole mount in situ hybridization, embryos were prepared as described previously [58], except that they were treated with 10 µg/ml proteinase K (QIAGEN) in phosphate-buffered saline, 0.1% Tween-20 (PBT) according to stage. Embryos were rinsed, postfixed and hybridized with digoxygenin-labeled probes in hybridization mix [50% formamide, 1.3× SSC, 5 mM EDTA, 50 µg/ml yeast RNA, 0.2% Tween 20, 0.5% 3-[(3-cholamidopropyl) dimethylammonio] propanesulfonate, and 100 µg/ml heparin] overnight at 65°C. After washing and blocking, embryos were incubated overnight with alkaline phosphatase-conjugated anti-digoxigenin antibody (1∶2000; Roche) in blocking buffer (2% blocking reagent [Roche]), 20% heat-inactivated lamb serum in 100 mM maleic acid, pH 7.5, 150 mM NaCl, and 0.1% Tween 20 [MABT]). After washing in TBST (Tris-buffered saline with 0.1% Tween-20) and (NTMT) 100 mm NaCl, 100 mm Tris-HCl, pH 9.5, 50 mm MgCl2, and 0.1% Tween −20, signals were developed with BM Purple AP Substrate (Roche). In situ terminal deoxynucleotidyltransferase-mediated dUTP-biotin nick end labeling (TUNEL) assay was performed according to the manufacturer's instructions (Roche). Whole mount immunohistochemistry for phospho-histone H3 (Upstate, 06-570) was performed with embryos fixed overnight in 4% paraformaldehyde in PBS [59], [60]. Embryos were dehydrated through a graded methanol series to 100%. Endogeneous peroxidase was inactivated by a one-hour treatment in 5% hydrogen peroxide in methanol. Embryos were rehydrated into 80%, 50% and 20% methanol in PBT (PBS+0.75% tween-20) and were washed for 10 minutes in PBT. Embryos were then incubated with blocking solution (PBT+10% goat serum) for one hour and incubated overnight at 4°C with anti phospho-Histone H3 antibody at a dilution of 1∶100 in blocking solution. Embryos were subsequently washed in PBT four times over one hour and then incubated overnight at 4°C with biotinylated anti-rabbit IgG (Vector Laboratories) at 1∶200 in blocking solution. After washing 4 times with PBT, embryos were then incubated with HRP-conjugated biotin-avidin as instructed by the manufacturer (Vector Laboratories, Vectastain ABC kit, PK-4000). Staining was developed with Sigma Fast DAB substrate (Sigma, D4418). Stained embryos were cleared in 80% glycerol and photographed with a Jenoptik ProgRes C3 camera attached on a Nikon SMZ 1500 stereomicroscope. Captured images were assembled by Helicon Focus software (Helicon Soft). Dissected embryos were stained for ß-galactosidase activity as described previously [27], [28], [30] with the following modification. Embryos were fixed with 2% paraformaldehyde and 0.2% glutaraldehyde in phosphate-buffered saline (PBS) for 10 minutes on ice and stained in PBS, pH 7.0, 2 mM MgCl_2_, 0.01% NP-40, and 0.02% sodium deoxycholate, 17.5 mM each K_3_Fe(CN)_6_ and K_4_Fe(CN)_6_ and 1 mg/ml 5-bromo-4-chloro-3-indolyl-β-d-galactoside (Roche). Stained embryos were cleared in 80% glycerol and PBS for photography.

### Bone and cartilage staining

Bones and cartilage of E19 embryos were stained with alizarin red and alcian blue as described [61]. Briefly, embryos were collected, fixed in 95% ethanol for 4–5 days and transferred to acetone for 3 days. The embryos were then rinsed with water and stained for 24 hours in 0.05% Alcian Blue in 20% glacial acetic acid in 95% ethanol. After washing in 95% ethanol for 2–3 days, soft tissues were dissolved in 1% KOH for 1 hour and stained in 0.75% Alizarin Red in 1% KOH for 4 hours. Stained embryos were kept in 20% glycerol/1% KOH until skeletons became clearly visible. Embryos were transferred through 50%, 80% and 100% glycerol for photography and storage.

### qRT–PCR

qRT–PCR analysis of *Shh*, *Gli1*, *Ptch1*, *Nkx2.1* and *Fgf8* expression was performed on E10.0 control (*Cdo^+/−^* or *Cdo^+/+^*) and *Cdo^−/−^* embryos. Embryonic forebrains were dissected out and transferred into 100 µl of RNAlater (Qiagen). Total RNA was purified using RNeasy Mini Kit (Qiagen). cDNA was synthesized with the Superscript III first strand synthesis system (Invitrogen). qPCR was performed using PerfeCta SYBR Green FastMix for iQ (Quanta bioscience) with Bio-Rad iCycler iQ5. Data were normalized to *Gapdh* levels and presented as fold change over control. qRT-PCR primers were from the Harvard PrimerBank (Primerbank IDs: *Gapdh*, 6679937a1; *Shh*, 21617861a1; *Gli1*, 6754002a1; *Ptch1*, 6679519a1; *Fgf8*, 22094093a1) and reference [62] (*Nkx2.1*).

## Supporting Information

Figure S1 Maternal blood alcohol levels. *Cdon^+/−^* females received IP injections of 2.9 g/kg (A) or 3.48 g/kg (B) ethanol (EtOH) in saline. EtOH was administered twice, at E7.0 and 4 hours later. Saline injections were used as a control. Values represent means ± S.E.M., n = 3 or 4 animals per point (A) and n = 3, 4 or 5 animals per point (B).
(TIF)

Figure S2 Loss of *Boc* and in utero ethanol (EtOH) exposure do not synergize to produce HPE. (A–D) Frontal views of E14.0 embryos. *Boc^−/−^* male mice were crossed with *Boc^+/−^* females and pregnant females were treated with saline or EtOH at E7.0. Embryos were collected at E14.0 and examined by whole mount. EtOH-treated *Boc^−/−^* embryos (D) did not display any external midline defects and were indistinguishable from untreated embryos or EtOH-treated *Boc^+/−^* embryos (A–C). Saline-treated *Boc^+/−^* embryos, n = 12; EtOH-treated *Boc^+/−^* embryos, n = 31; saline-treated *Boc^−/−^* embryos, n = 13; EtOH-treated *Boc^−/−^* embryos, n = 28.
(TIF)

Figure S3 Early *Six3* expression was not affected by loss of *Cdon* or ethanol (EtOH) treatment. Whole mount in situ hybridization analysis of *Six3* expression in embryos of the indicated genotype and treatment at the 8 to 9-somite stage (lateral views). *Six3* is expressed in the ventral forebrain. Scale bar, 250 µm. N = 2 embryos for each condition except for ethanol-treated *Cdon^−/−^* embryos where n = 3.
(TIF)

Figure S4 Apoptosis and cell proliferation in the anterior neural plate in wild-type and *Cdon^−/−^* embryos after saline or ethanol (EtOH) treatment. (A–E) Apoptosis is increased in the anterior neural folds (ANF) of EtOH-treated embryos at E8.0 (4–6 somites), independent of *Cdon* genotype. However, there is little apoptosis in the midline region and EtOH had no effect on this. (A) Mean number of TUNEL-positive cells in the ANF or the midline ± S.D. Ethanol-treated *Cdon^−/−^* embryos, n = 9; all other conditions, n = 4 each. (B–E) Micrographs of E8.0 embryos analyzed by in situ TUNEL assay (dorsal views). Scale bar = 250 µm. (F–J) Cell proliferation is not affected in the ANF or midline at E8.0 (4–6 somites) in wild-type and *Cdon^−/−^* embryos plus or minus EtOH treatment. (F) Mean number of phospho-histone H3-positive cells in 0.0625 mm^2^ of the ANF or midline ± S.D. EtOH-treated *Cdon^−/−^* embryos, n = 7; all other conditions, n = 5 each. (G–J) Micrographs of E8.0 embryos stained with antibody to phospho-histone H3 (dorsal views). Scale bar, 250 µm.
(TIF)

Figure S5 The expression patterns of the migrating neural crest cell markers *Msx1* and *Msx2* were not affected by loss of *Cdon* or ethanol (EtOH) treatment. Whole mount in situ of hybridization analysis of *Msx1* expression (A–D) and *Msx2* expression (E–H) at the 32 to 36-somite stage (frontal views). Scale bar, 250 µm. N = 2 embryos for each condition for both *Msx1* and *Msx2* except for EtOH-treated *Cdon^−/−^* embryos where n = 5 for *Msx1* and n = 6 for *Msx2*.
(TIF)

Figure S6 Analysis of *Shh*, *Gli1*, *Ptch1*, *Nkx2.1* and *Fgf8* expression in the embryonic forebrain at E10.0. Microdissected forebrains were analyzed by quantitative RT-PCR (qRT-PCR). Each symbol corresponds to an individual embryo; multiple litters were used. qRT-PCR signals were normalized to *Gapdh* expression and the means for Control (*Cdon^+/+^* or *Cdon^+/−^*) Saline embryos were set to 1.0. Bars represent S.E.M. *p<0.05, **p<0.005, ***p<0.0005 by Student's *t*-test. In some cases, (*Ptch1* and *Nkx2.1*), EtOH-treated *Cdon^−/−^* embryonic forebrains were significantly different from all three other (control) conditions, whereas in other cases these were significantly different from two (*Shh*, *Fgf8*) or one (*Gli1*) other control condition. Note that the three control conditions do not display defects in expression pattern by in situ hybridization (see Figure 4, Figure 5, Table 2, and Table 3). Furthermore, the EtOH-treated *Cdon^−/−^* embryonic forebrains showed the lowest mean expression values and lowest individual expression values for all genes examined. As noted in the text, it is likely that the qRT-PCR results underestimate the reduction in expression of these genes in the most affected region of EtOH-treated *Cdon^−/−^* embryos (the rostroventral midline) because more caudal and lateral forebrain structures, where changes in expression are not obvious, were by necessity included in the dissected region of the embryos. Note also that the generally greater range of expression values for EtOH-treated *Cdon^−/−^* embryos is consistent with the penetrance and expressivity of HPE phenotypes seen in such embryos. These latter points are highly likely to be the cause of the lack of statistical significance of EtOH-treated *Cdon^−/−^* embryos against every control condition for *Shh*, *Fgf8* and *Gli1*.
(TIF)

Figure S7 Immunostaining of E10.0 embryo sections of the indicated genotype treated in utero with ethanol (EtOH) or saline (control) for expression of markers of dorsoventral patterning of the neural tube. (A–D) FOXA2. (E–H) NKX2.2. (I–L) NKX6.1. (M–P) PAX6. (Q–T) PAX7. (U, V) Quantification of numbers of FOXA2^+^ and NKX2.2^+^ cells, respectively. (W, X, Y) Quantification of NKX6.1^+^, PAX6^+^ and PAX7^+^ cells, respectively, was done by measuring the size of the expression domain of each individual marker relative to the size of the entire neural tube. Values are means ± S.D, n = 3–5. Note that neural tube patterning was unperturbed by loss of *Cdon* and/or EtOH treatment.
(TIF)

Figure S8 Loss of *Cdon* and in utero ethanol (EtOH) exposure do not synergize to produce HPE when EtOH is administered at E8.0. (A–D) Frontal views of E14.0 embryos. *Cdon^+/−^* male mice were crossed with *Cdon^+/−^* females and pregnant females were treated with saline or EtOH at E8.0. Embryos were collected at E14.0 and examined by whole mount. Unlike embryos treated at E7.0, *Cdon^−/−^* embryos treated at E8.0 (D) did not display any external midline defects and were indistinguishable from untreated embryos or EtOH-treated *Cdon^+/+^* embryos (A–C). Saline-treated *Cdon^+/+^* embryos, n = 15; EtOH-treated *Cdon^+/+^* embryos, n = 11; saline-treated *Cdon^−/−^* embryos, n = 14; EtOH-treated *Cdon^−/−^* embryos, n = 13.
(TIF)

Table S1 Offspring of intercrosses of *Cdon^+/−^* mice with pregnant females treated with ethanol or saline at E7.0. Note that the embryos with severe HPE found at E10.0 are not recovered at E14.0, and analysis at E11.0 revealed embryos in the process of resorption in the *Cdon^−/−^* plus ethanol group. We therefore assume that these latter embryos include all those with severe HPE. However, natural deviation from Mendelian inheritance is inherent with the numbers of embryos analyzed and therefore does not allow for statistical proof of their absence. * 15 of 111 (13.5%) ethanol-treated E10.0 *Cdon^−/−^* embryos displayed alobar HPE (Figure 1). ** 1 of 22 (4.5%) saline-treated *Cdon^−/−^* embryos displayed external features of HPE. *** 13 of 18 (72.2%) ethanol-treated *Cdon^−/−^* embryos displayed external features of HPE (Figure 1).
(DOC)

Table S2 Number of somites in saline- and ethanol-treated *Cdon^+/+^*, *Cdon^+/−^* and *Cdon^−/−^* embryos at E8.0, E9.0 and E10.0. *Cdon^+/−^* mice were intercrossed and pregnant females treated with ethanol or saline control at E7.0. Embryos were collected at the indicated stage, genotyped and somites counted. Note that ethanol-treated embryos at each stage have between 1 and 3 fewer somite pairs than saline controls at each stage, independent of genotype. *, Somite numbers of ethanol-treated embryos are different from those of saline-treated embryos of the same genotype at the same stage, p<0.05 by Student's t-test. Somite numbers of *Cdon^+/+^*, *Cdon^+/−^* and *Cdon^−/−^* embryos are not significantly different from each other within the ethanol-treated or saline-treated groups at any stage.
(DOC)
