# Supplementary figures and images for: Cdon Mutation and Fetal Ethanol Exposure Synergize to Produce Midline Signaling Defects and Holoprosencephaly Spectrum Disorders in Mice
Source: PLoS Genet. 2012 Oct 11;8(10):e1002999. doi: 10.1371/journal.pgen.1002999 (PMC3469434; doi:10.1371/journal.pgen.1002999)

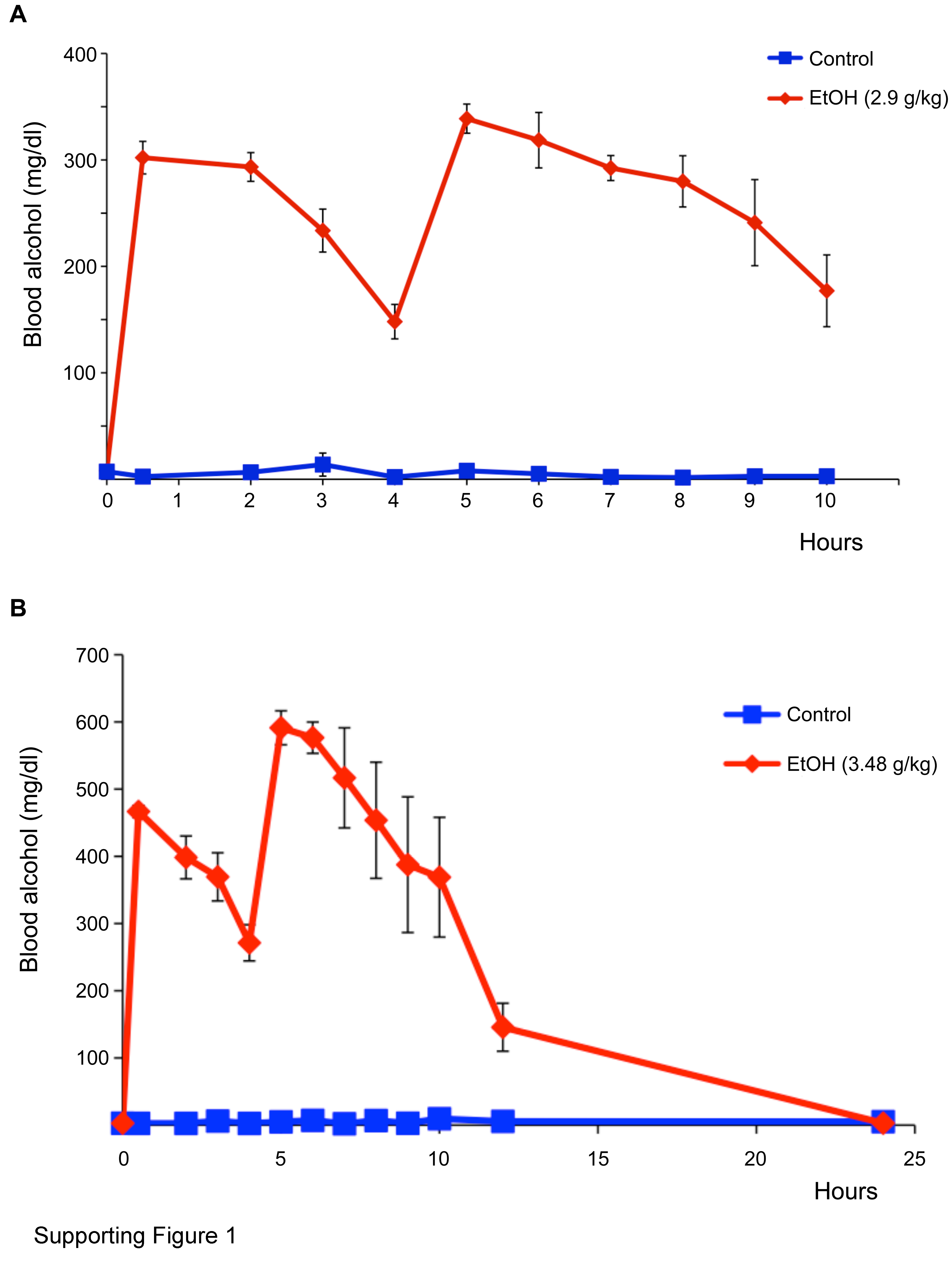

Supplement: Figure S1 — Maternal blood alcohol levels. Cdon+/− females received IP injections of 2.9 g/kg (A) or 3.48 g/kg (B) ethanol (EtOH) in saline. EtOH was administered twice, at E7.0 and 4 hours later. Saline injections were used as a control. Values represent means ± S.E.M., n = 3 or 4 animals per point (A) and n = 3, 4 or 5 animals per point (B). (TIF) [file pgen.1002999.s001.tif]

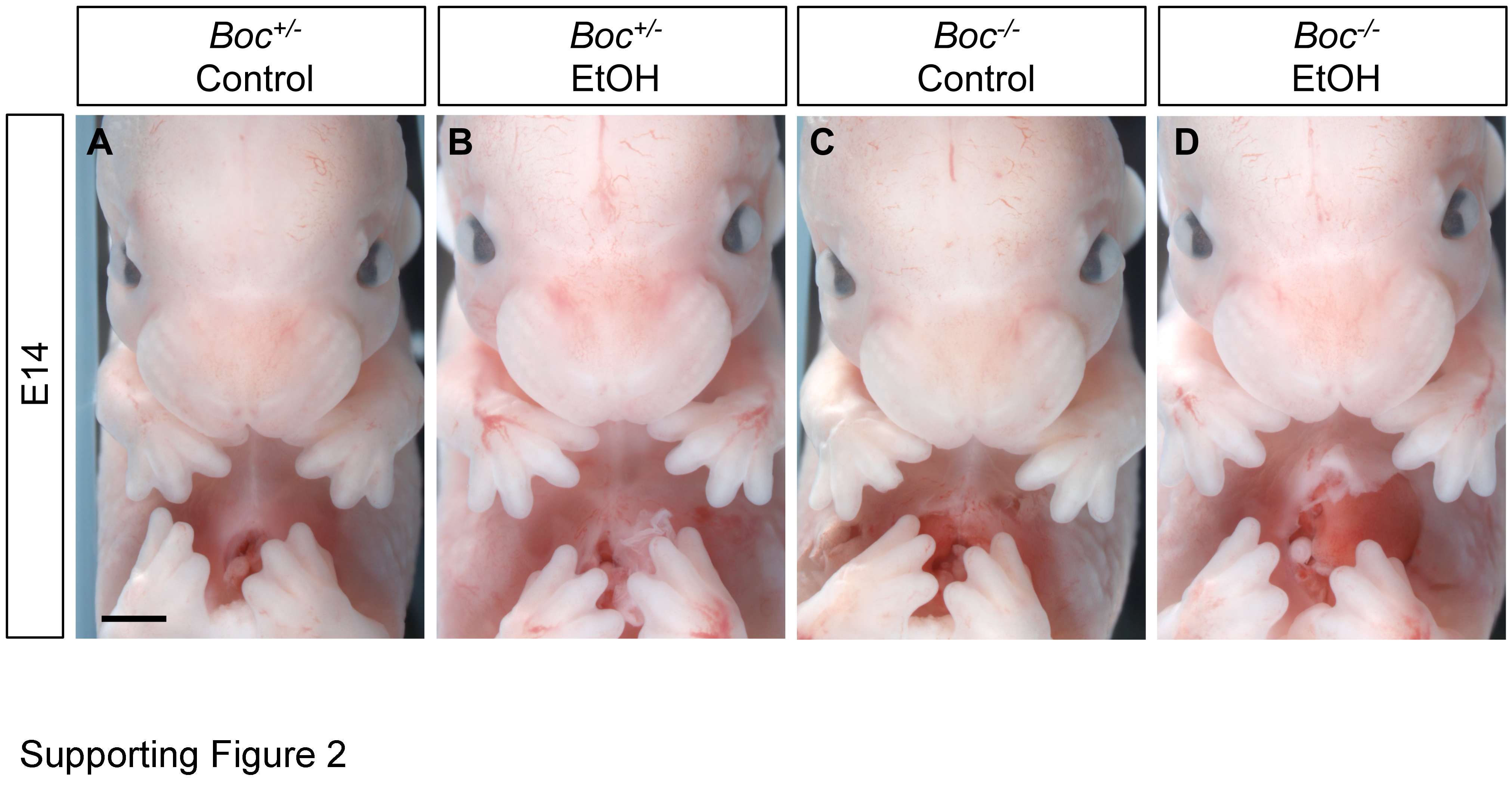

Supplement: Figure S2 — Loss of Boc and in utero ethanol (EtOH) exposure do not synergize to produce HPE. (A–D) Frontal views of E14.0 embryos. Boc−/− male mice were crossed with Boc+/− females and pregnant females were treated with saline or EtOH at E7.0. Embryos were collected at E14.0 and examined by whole mount. EtOH-treated Boc−/− embryos (D) did not display any external midline defects and were indistinguishable from untreated embryos or EtOH-treated Boc+/− embryos (A–C). Saline-treated Boc+/− embryos, n = 12; EtOH-treated Boc+/− embryos, n = 31; saline-treated Boc−/− embryos, n = 13; EtOH-treated Boc−/− embryos, n = 28. (TIF) [file pgen.1002999.s002.tif]

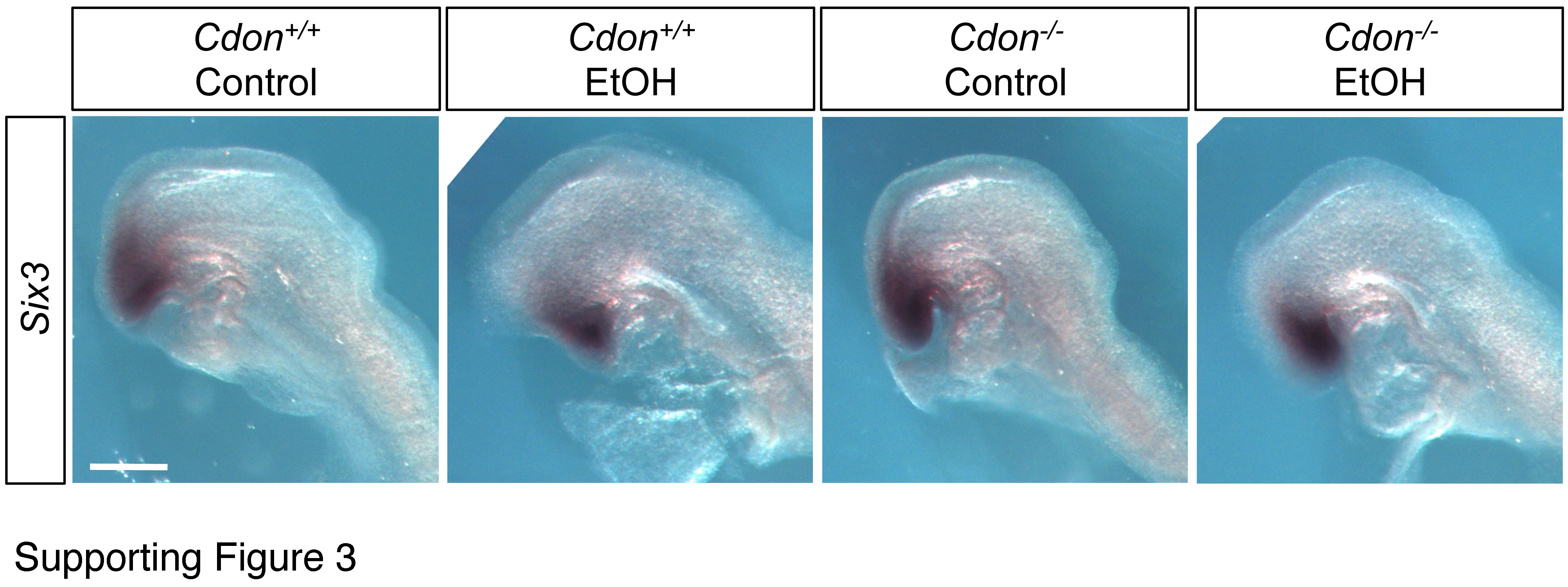

Supplement: Figure S3 — Early Six3 expression was not affected by loss of Cdon or ethanol (EtOH) treatment. Whole mount in situ hybridization analysis of Six3 expression in embryos of the indicated genotype and treatment at the 8 to 9-somite stage (lateral views). Six3 is expressed in the ventral forebrain. Scale bar, 250 µm. N = 2 embryos for each condition except for ethanol-treated Cdon−/− embryos where n = 3. (TIF) [file pgen.1002999.s003.tif]

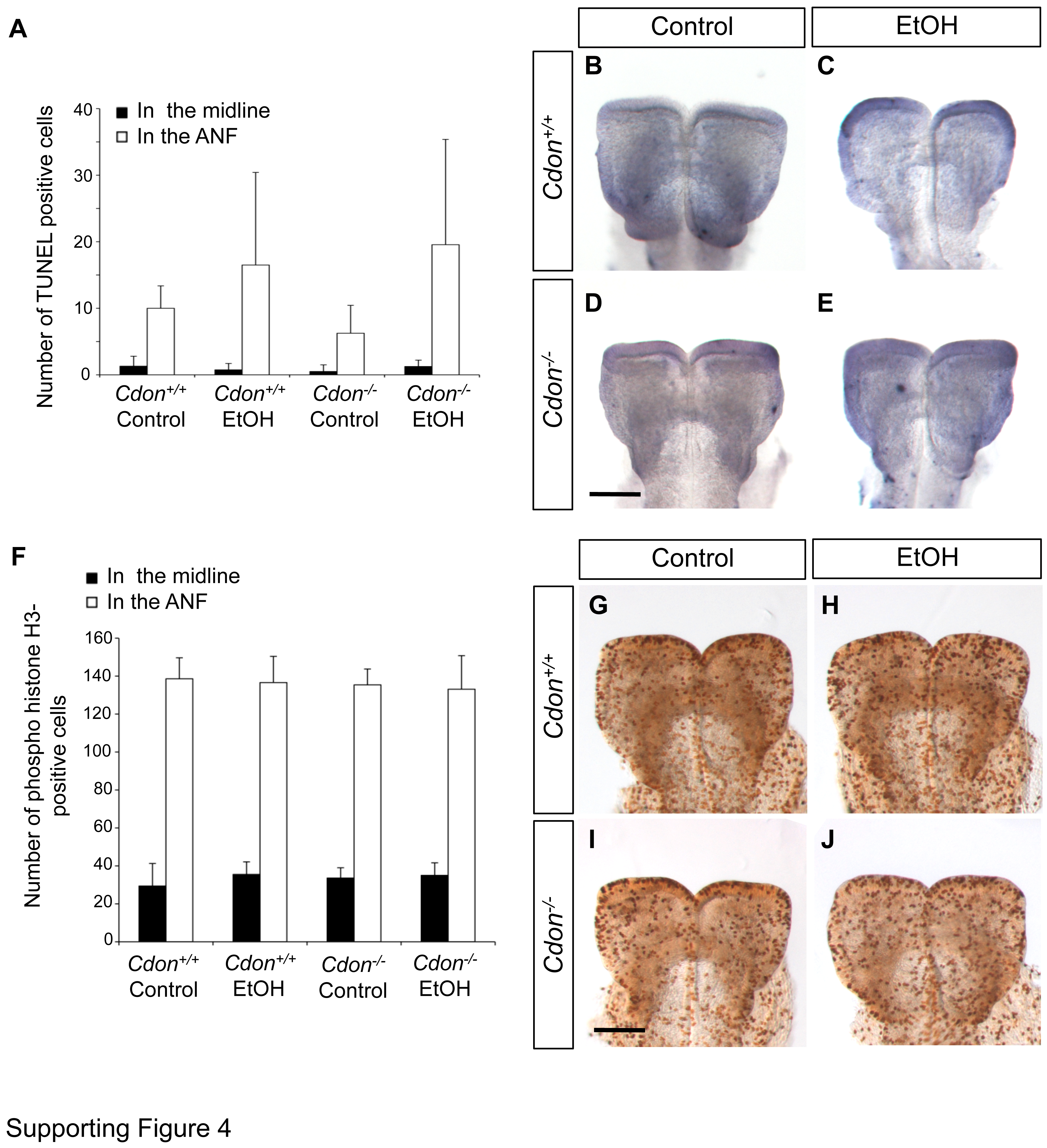

Supplement: Figure S4 — Apoptosis and cell proliferation in the anterior neural plate in wild-type and Cdon−/− embryos after saline or ethanol (EtOH) treatment. (A–E) Apoptosis is increased in the anterior neural folds (ANF) of EtOH-treated embryos at E8.0 (4–6 somites), independent of Cdon genotype. However, there is little apoptosis in the midline region and EtOH had no effect on this. (A) Mean number of TUNEL-positive cells in the ANF or the midline ± S.D. Ethanol-treated Cdon−/− embryos, n = 9; all other conditions, n = 4 each. (B–E) Micrographs of E8.0 embryos analyzed by in situ TUNEL assay (dorsal views). Scale bar = 250 µm. (F–J) Cell proliferation is not affected in the ANF or midline at E8.0 (4–6 somites) in wild-type and Cdon−/− embryos plus or minus EtOH treatment. (F) Mean number of phospho-histone H3-positive cells in 0.0625 mm2 of the ANF or midline ± S.D. EtOH-treated Cdon−/− embryos, n = 7; all other conditions, n = 5 each. (G–J) Micrographs of E8.0 embryos stained with antibody to phospho-histone H3 (dorsal views). Scale bar, 250 µm. (TIF) [file pgen.1002999.s004.tif]

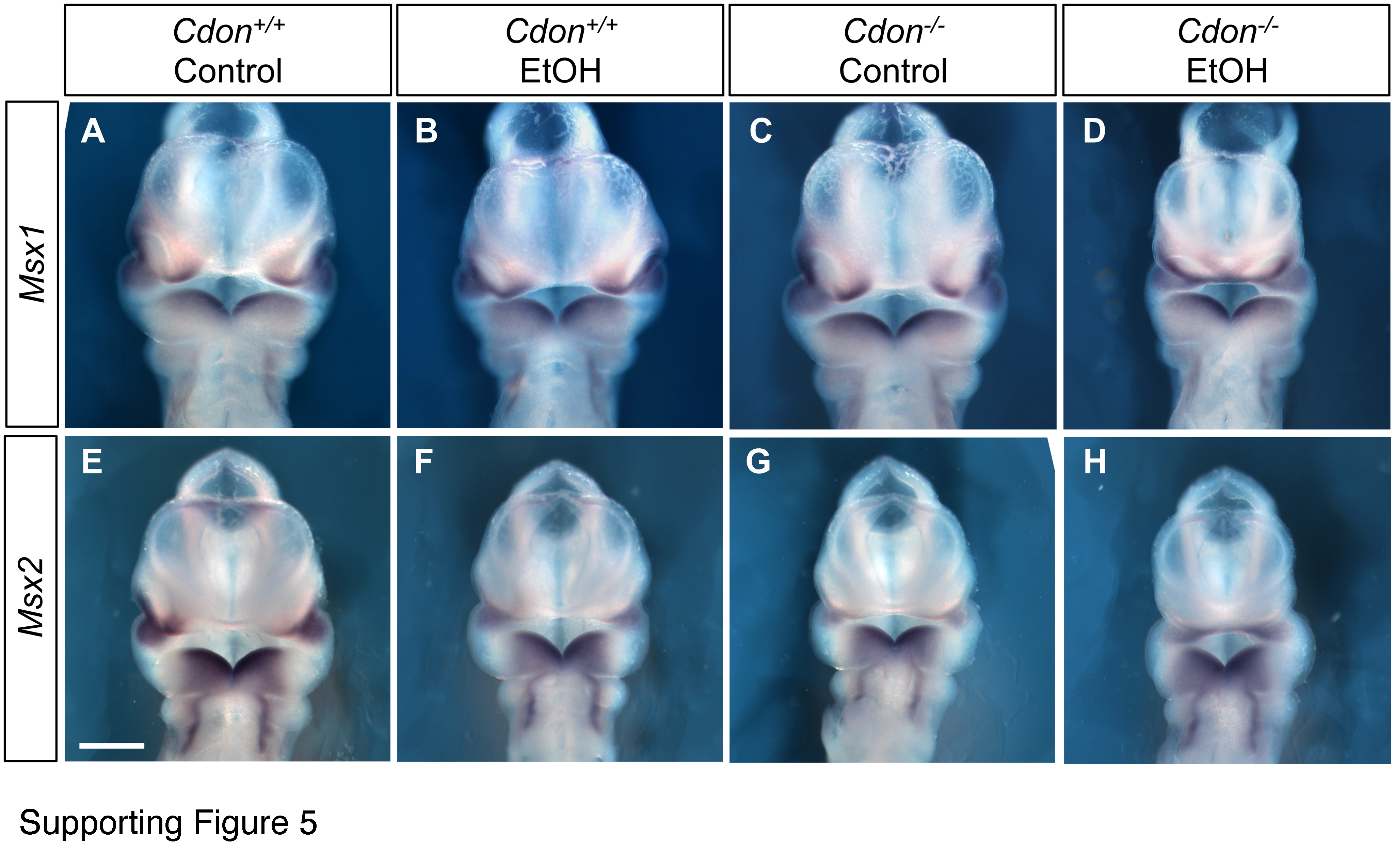

Supplement: Figure S5 — The expression patterns of the migrating neural crest cell markers Msx1 and Msx2 were not affected by loss of Cdon or ethanol (EtOH) treatment. Whole mount in situ of hybridization analysis of Msx1 expression (A–D) and Msx2 expression (E–H) at the 32 to 36-somite stage (frontal views). Scale bar, 250 µm. N = 2 embryos for each condition for both Msx1 and Msx2 except for EtOH-treated Cdon−/− embryos where n = 5 for Msx1 and n = 6 for Msx2. (TIF) [file pgen.1002999.s005.tif]

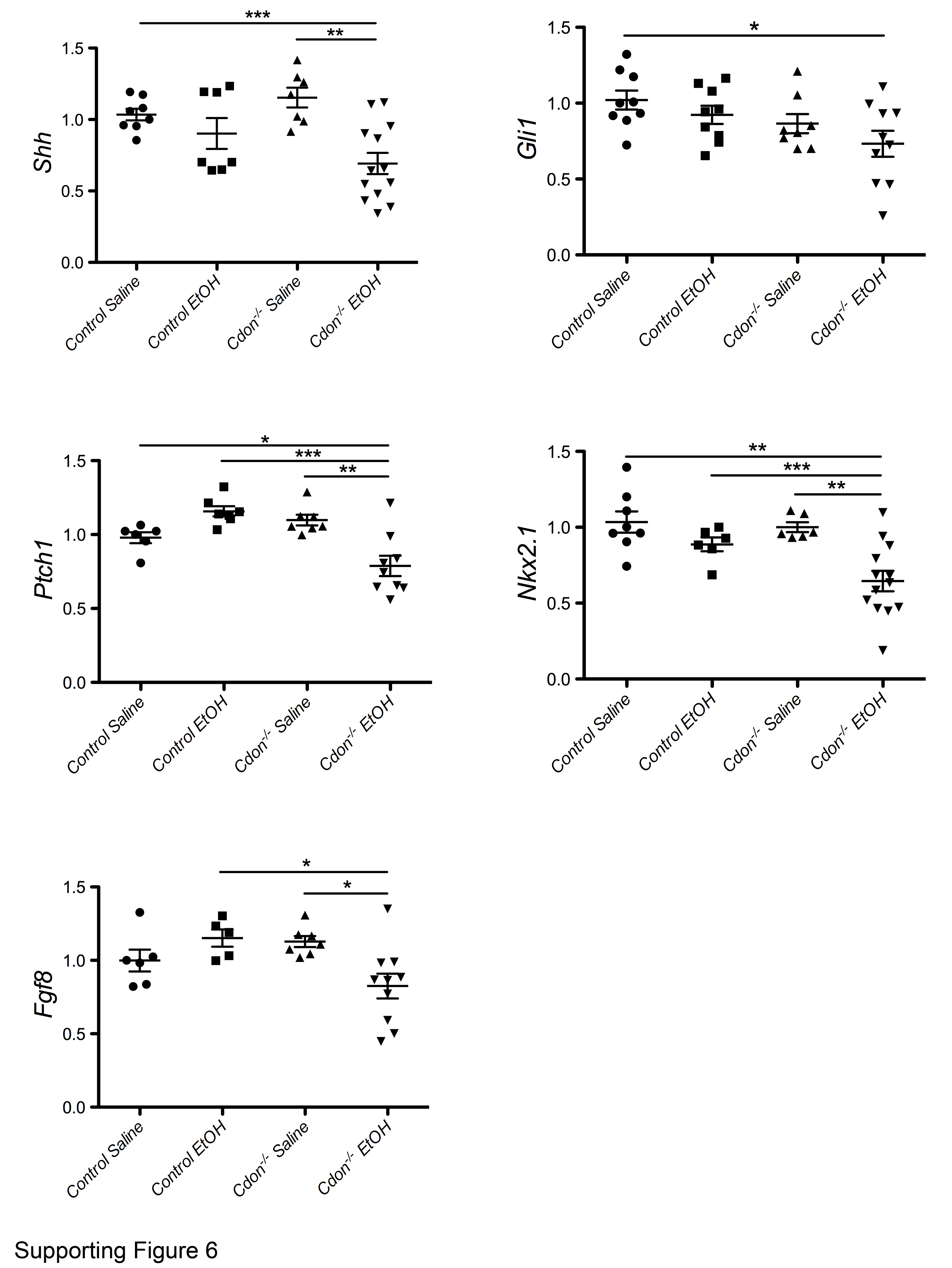

Supplement: Figure S6 — Analysis of Shh, Gli1, Ptch1, Nkx2.1 and Fgf8 expression in the embryonic forebrain at E10.0. Microdissected forebrains were analyzed by quantitative RT-PCR (qRT-PCR). Each symbol corresponds to an individual embryo; multiple litters were used. qRT-PCR signals were normalized to Gapdh expression and the means for Control (Cdon+/+ or Cdon+/−) Saline embryos were set to 1.0. Bars represent S.E.M. *p<0.05, **p<0.005, ***p<0.0005 by Student's t-test. In some cases, (Ptch1 and Nkx2.1), EtOH-treated Cdon−/− embryonic forebrains were significantly different from all three other (control) conditions, whereas in other cases these were significantly different from two (Shh, Fgf8) or one (Gli1) other control condition. Note that the three control conditions do not display defects in expression pattern by in situ hybridization (see Figure 4, Figure 5, Table 2, and Table 3). Furthermore, the EtOH-treated Cdon−/− embryonic forebrains showed the lowest mean expression values and lowest individual expression values for all genes examined. As noted in the text, it is likely that the qRT-PCR results underestimate the reduction in expression of these genes in the most affected region of EtOH-treated Cdon−/− embryos (the rostroventral midline) because more caudal and lateral forebrain structures, where changes in expression are not obvious, were by necessity included in the dissected region of the embryos. Note also that the generally greater range of expression values for EtOH-treated Cdon−/− embryos is consistent with the penetrance and expressivity of HPE phenotypes seen in such embryos. These latter points are highly likely to be the cause of the lack of statistical significance of EtOH-treated Cdon−/− embryos against every control condition for Shh, Fgf8 and Gli1. (TIF) [file pgen.1002999.s006.tif]

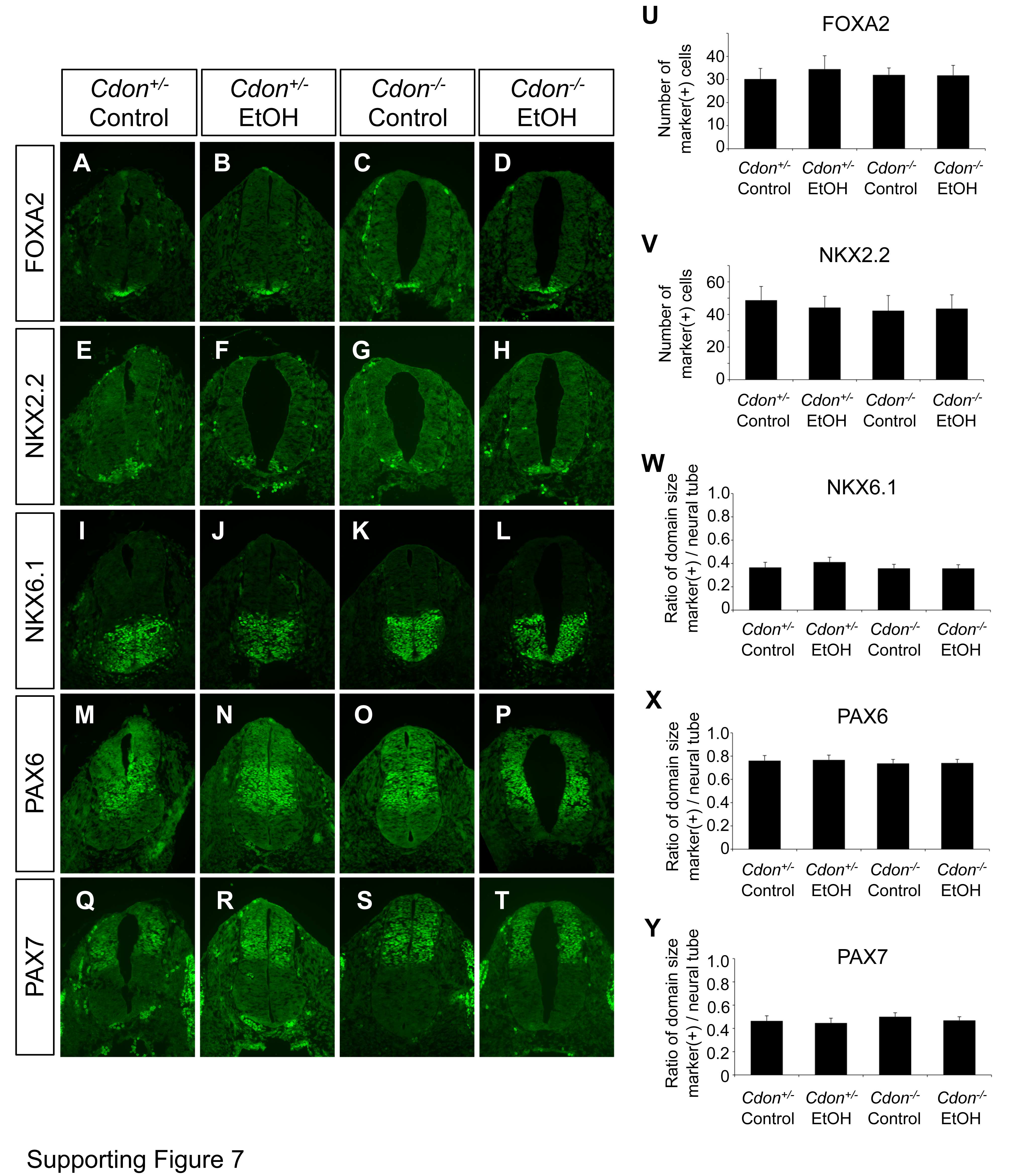

Supplement: Figure S7 — Immunostaining of E10.0 embryo sections of the indicated genotype treated in utero with ethanol (EtOH) or saline (control) for expression of markers of dorsoventral patterning of the neural tube. (A–D) FOXA2. (E–H) NKX2.2. (I–L) NKX6.1. (M–P) PAX6. (Q–T) PAX7. (U, V) Quantification of numbers of FOXA2+ and NKX2.2+ cells, respectively. (W, X, Y) Quantification of NKX6.1+, PAX6+ and PAX7+ cells, respectively, was done by measuring the size of the expression domain of each individual marker relative to the size of the entire neural tube. Values are means ± S.D, n = 3–5. Note that neural tube patterning was unperturbed by loss of Cdon and/or EtOH treatment. (TIF) [file pgen.1002999.s007.tif]

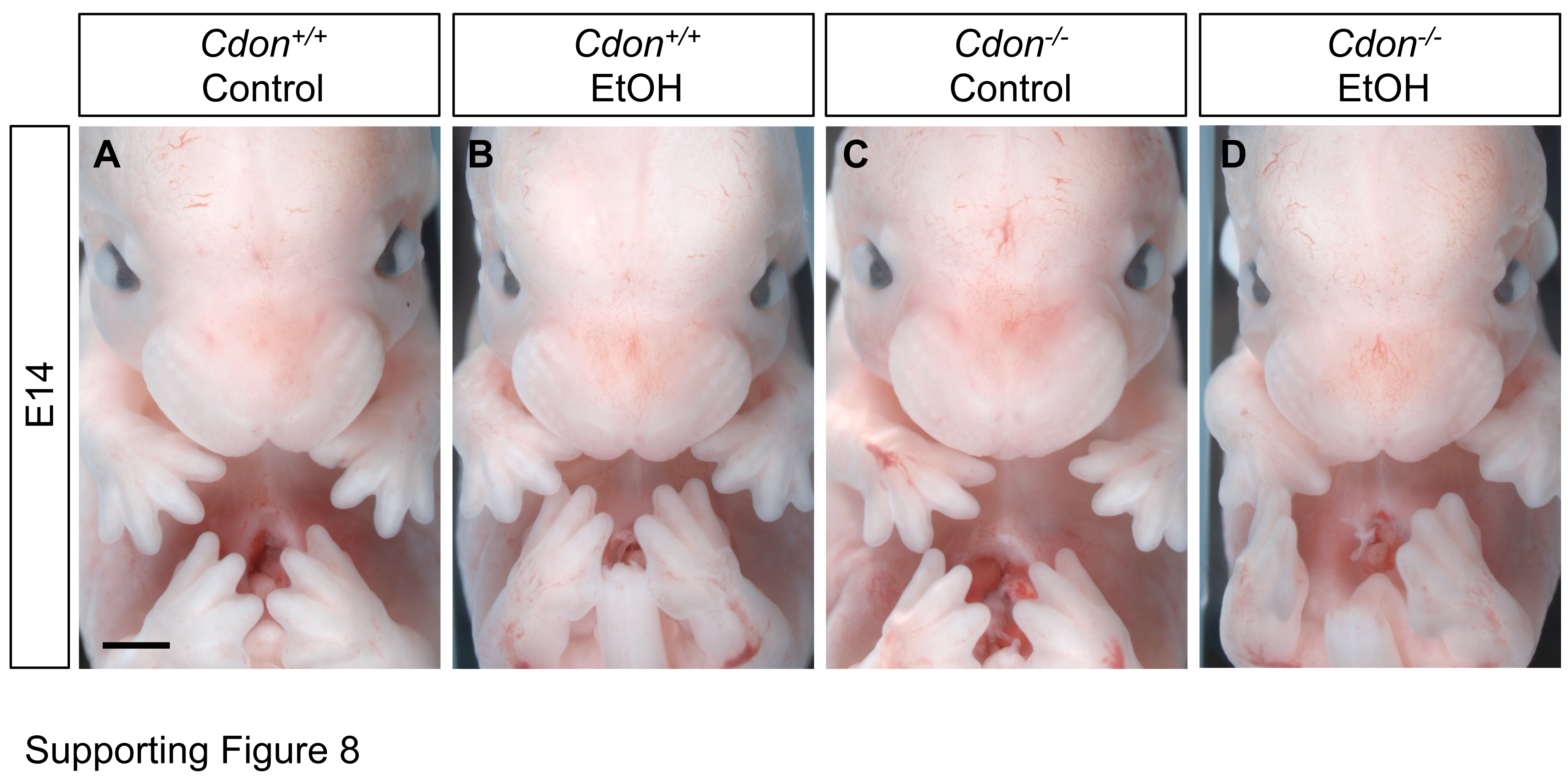

Supplement: Figure S8 — Loss of Cdon and in utero ethanol (EtOH) exposure do not synergize to produce HPE when EtOH is administered at E8.0. (A–D) Frontal views of E14.0 embryos. Cdon+/− male mice were crossed with Cdon+/− females and pregnant females were treated with saline or EtOH at E8.0. Embryos were collected at E14.0 and examined by whole mount. Unlike embryos treated at E7.0, Cdon−/− embryos treated at E8.0 (D) did not display any external midline defects and were indistinguishable from untreated embryos or EtOH-treated Cdon+/+ embryos (A–C). Saline-treated Cdon+/+ embryos, n = 15; EtOH-treated Cdon+/+ embryos, n = 11; saline-treated Cdon−/− embryos, n = 14; EtOH-treated Cdon−/− embryos, n = 13. (TIF) [file pgen.1002999.s008.tif]
