# Supplementary material for: Cdon Mutation and Fetal Ethanol Exposure Synergize to Produce Midline Signaling Defects and Holoprosencephaly Spectrum Disorders in Mice
Source: PLoS Genet. 2012 Oct 11;8(10):e1002999. doi: 10.1371/journal.pgen.1002999 (PMC3469434; doi:10.1371/journal.pgen.1002999)
Supplement: Table S1 — Offspring of intercrosses of Cdon+/− mice with pregnant females treated with ethanol or saline at E7.0. Note that the embryos with severe HPE found at E10.0 are not recovered at E14.0, and analysis at E11.0 revealed embryos in the process of resorption in the Cdon−/− plus ethanol group. We therefore assume that these latter embryos include all those with severe HPE. However, natural deviation from Mendelian inheritance is inherent with the numbers of embryos analyzed and therefore does not allow for statistical proof of their absence. * 15 of 111 (13.5%) ethanol-treated E10.0 Cdon−/− embryos displayed alobar HPE (Figure 1). ** 1 of 22 (4.5%) saline-treated Cdon−/− embryos displayed external features of HPE. *** 13 of 18 (72.2%) ethanol-treated Cdon−/− embryos displayed external features of HPE (Figure 1). (DOC) [file pgen.1002999.s009.doc]

**Table S1.** Offspring of intercrosses of *Cdon^+/-^* mice with pregnant females treated with ethanol or saline at E7.0

| **Number of embryos of genotype:** | | | | | |
| --- | --- | --- | --- | --- | --- |
| **Stage** | **Treatment** | **No. living embryos** | ***Cdon^+/+^*** | ***Cdon^+/-^*** | ***Cdon^-/-^*** |
|  |  |  |  |  |  |
| E8.0 | Saline | 167 (91.8%) | 42 (25.1%) | 81 (48.5%) | 44 (26.3%) |
|  | Ethanol | 393 (93.6%) | 111 (28.2%) | 186 (47.3%) | 96 (24.4%) |
|  |  |  |  |  |  |
| E9.0 | Saline | 96 (96.0%) | 29 (30.2%) | 44 (45.8%) | 23 (24.0%) |
|  | Ethanol | 75 (100%) | 16 (21.3%) | 41 (54.7%) | 18 (24.0%) |
|  |  |  |  |  |  |
| E10.0 | Saline | 134 (95.0%) | 49 (36.6%) | 50 (37.3%) | 35 (26.1%) |
|  | Ethanol | 405 (94.8%) | 115 (28.4%) | 179 (44.2%) | 111 (27.4%)* |
|  |  |  |  |  |  |
| E14.0 | Saline | 106 (94.6%) | 36 (34%) | 48 (45.3%) | 22 (20.8%)** |
|  | Ethanol | 63 (84%) | 12 (19.0%) | 33 (52.4%) | 18 (28.6%)*** |

* 15 of 111 (13.5%) ethanol-treated E10.0 *Cdon^-/-^* embryos displayed alobar HPE (Figure 1).

** 1 of 22 (4.5%) saline-treated *Cdon^-/-^* embryos displayed external features of HPE.

*** 13 of 18 (72.2%) ethanol-treated *Cdon^-/-^* embryos displayed external features of HPE (Figure 1).

The embryos with severe HPE found at E10.0 are not recovered at E14.0, and analysis at E11.0 revealed embryos in the process of resorption in the *Cdon^-/-^* plus ethanol group. We therefore assume that these latter embryos include all those with severe HPE. However, natural deviation from Mendelian inheritance is inherent with the numbers of embryos analyzed and therefore does not allow for statistical proof of their absence.
