# Supplementary material for: Cdon Mutation and Fetal Ethanol Exposure Synergize to Produce Midline Signaling Defects and Holoprosencephaly Spectrum Disorders in Mice
Source: PLoS Genet. 2012 Oct 11;8(10):e1002999. doi: 10.1371/journal.pgen.1002999 (PMC3469434; doi:10.1371/journal.pgen.1002999)
Supplement: Table S2 — Number of somites in saline- and ethanol-treated Cdon+/+, Cdon+/− and Cdon−/− embryos at E8.0, E9.0 and E10.0. Cdon+/− mice were intercrossed and pregnant females treated with ethanol or saline control at E7.0. Embryos were collected at the indicated stage, genotyped and somites counted. Note that ethanol-treated embryos at each stage have between 1 and 3 fewer somite pairs than saline controls at each stage, independent of genotype. *, Somite numbers of ethanol-treated embryos are different from those of saline-treated embryos of the same genotype at the same stage, p<0.05 by Student's t-test. Somite numbers of Cdon+/+, Cdon+/− and Cdon−/− embryos are not significantly different from each other within the ethanol-treated or saline-treated groups at any stage. (DOC) [file pgen.1002999.s010.doc]

**Table S2.** Number of somites in saline- and ethanol-treated *Cdon^+/+^*, *Cdon^+/-^* and *Cdon^-/-^* embryos at E8.0, E9.0 and E10.0

| **Number of Somites** | | | | |
| --- | --- | --- | --- | --- |
| **Stage** | **Treatment** | ***Cdon^+/+^*** | ***Cdon^+/-^*** | ***Cdon^-/-^*** |
|  |  |  |  |  |
| E8.0 | Saline (n=167) | 4.0 ± 2.5 | 4.2 ± 2.4 | 4.3 ± 2.1 |
|  | Ethanol (n=393) | 2.3 ± 2.1* | 2.6 ± 2.3* | 2.3 ± 2.3* |
|  |  |  |  |  |
| E9.0 | Saline (n=96) | 18.8 ± 4.1 | 18.2 ± 5.1 | 18.7 ± 3.0 |
|  | Ethanol (n=75) | 15.9 ± 3.3* | 16.0 ± 3.9* | 16.6 ± 4.0* |
|  |  |  |  |  |
| E10.0 | Saline (n=134) | 32.1 ± 2.8 | 32.3 ± 2.5 | 32.7 ± 2.3 |
|  | Ethanol (n=405) | 31.1 ± 2.6 | 31.0 ± 2.7* | 30.9 ± 2.9* |
|  |  |  |  |  |

*Cdon^+/-^* mice were intercrossed and pregnant females treated with ethanol or saline control at E7.0. Embryos were collected at the indicated stage, genotyped and somites counted. Note that ethanol-treated embryos at each stage have between 1 and 3 fewer somite pairs than saline controls at each stage, independent of genotype.

*, Somite numbers of ethanol-treated embryos are different from those of saline-treated embryos of the same genotype at the same stage, p < 0.05 by Student’s t-test. Somite numbers of *Cdon^+/+^*, *Cdon^+/-^* and *Cdon^-/-^* embryos are not significantly different from each other within the ethanol-treated or saline-treated groups at any stage.
